# Seasonal changes in the distributions of fish and zooplankton across the Barents Sea Polar Front

**DOI:** 10.1371/journal.pone.0348949

**Published:** 2026-05-11

**Authors:** Einat Sandbank, Malin Daase, Paul E. Renaud, Sünnje L. Basedow, Maxime Geoffroy

**Affiliations:** 1 Centre for Fisheries Ecosystems Research, Fisheries and Marine Institute of Memorial University of Newfoundland, St. John’s, Canada; 2 Department of Arctic Biology, The University Centre in Svalbard, Longyearbyen, Norway; 3 Department of Arctic and Marine Biology, Faculty of Biosciences, Fisheries and Economics, UiT The Arctic University of Norway, Tromsø, Norway; 4 Akvaplan-niva, Tromsø, Norway; University of Messina, ITALY

## Abstract

Oceanic fronts are often characterized by high primary productivity and increased localized levels of species diversity and biomass; this is due in part to the mixing of distinct water masses as well as the aggregation of organisms by ocean currents. How these interactions between the physical and chemical parameters of the Barents Sea Polar Front impact pelagic ecology are poorly understood, particularly across seasons. By combining findings from continuous acoustic and hydrographic recordings with discrete sampling, we measured how hydrographic changes associated with the Barents Sea Polar Front modify the spatial distribution and biomass of fish and zooplankton across three seasons (spring, winter, and summer). We found the influence of the Polar Front on the ecology of pelagic organisms to be highly dependent on season, with spring having the strongest correlation between hydrographic parameters and species distribution. The Front’s influence on zooplankton and fishes differed considerably, as zooplankton and fish biomass often peaked at opposing ends of our survey transects, and in spring resulted in a zooplankton refuge from grazing fishes. The importance of seasonal sea ice was evident, and both the spring ice melt and new winter ice were strongly correlated with species distribution and in spring also biodiversity. Abiotic variables, and in particular temperature, were found to be the strongest predictors of pelagic fish and macrozooplankton biomass in all seasons, although in winter a greater influence by biotic variables was observed. While oceanic fronts are traditionally considered hotspots of biodiversity, the strong seasonal changes in the structure of the Barents Sea Polar Front create everchanging conditions where high biodiversity and pelagic biomass do not persist year-round. Our findings highlight the importance of considering seasonality in management decisions, and the need for increased in-situ winter data collection.

## Introduction

Oceanic fronts occur at the junction of distinct water masses. These widely studied regions play important roles in the global transfer and circulation of heat, salinity, oxygen and nutrients [1–4]. Fronts are, thus, often locations of high productivity and biodiversity [5,6] as the intersection of water masses that form oceanic fronts enhance nutrient recycling and thereby create localized blooms of phytoplankton [4,5]. This higher level of primary productivity attracts and aggregates grazing zooplankton and their predators [6,7]. Fronts are shaped by a variety of processes [1], and thus their size, longevity, magnitude, and geographical extent vary greatly [3,4,7,8]. Variability in these dynamics can also exist within larger oceanic fronts, such as the Barents Sea Polar Front.

The Barents Sea Polar Front acts as a boundary dividing the Barents Sea into a boreal southern region and an Arctic northern region [3,9,10] This semi-static boundary extends through the majority of the Barents Sea, and is important to the circulation and ventilation of the Arctic Ocean [3,11,12].The majority of the region south of the Front is ice-free throughout the year as Atlantic waters entering the Barents Sea, as well as local climate, limit the extent of winter sea ice towards the south [3,12,13]. The western segment of the Front is relatively stable at the 200–250 m isobath [14,15] and results in the presence of distinct water masses [3,9,15]. In contrast, the eastern portion of the Front is ephemeral and less pronounced, as shallower and more uniform seafloor facilitates mixing, impeding the persistence of unique water masses [15]. Despite many years of research, the intricacies of the physical structure of the Barents Sea Polar Front and its influence on pelagic organisms have not been explored in detail, especially across seasons.

The physical and chemical differences in waters north and south of the Polar Front create distinct conditions where the level of primary productivity and timing of phytoplankton blooms differs between the two regions [16–18]. North of the Polar Front, ecological processes are primarily controlled by seasonal ice formation and melt [19,20]. The timing of the spring bloom is often linked to the timing of seasonal ice melt and thus generally occurs slightly later north of the Polar Front where sea ice persists longer [21,22]. However, recent studies have shown that the spring bloom is not only initiated by open water and the stratification created by melt water but can begin earlier, while under seasonal sea ice [23].

The Barents Sea is highly productive and supports some of the world’s commercially important finfish stocks, including cod (*Gadus morhua),* haddock (*Melanogrammus aeglefinus*), capelin (*Mallotus villosus*) [24,25], as well as commercially important shellfish fisheries such as northern shrimp (*Pandalus borealis*) and the introduced snow crab (*Chionoecetes opilio*) and red king crab (*Paralithodes camtschaticu*) [26]. South of the Polar Front, the Barents Sea is described as an extension of the Norwegian Sea and is characterized by boreal and temperate species where haddock, Atlantic cod, capelin and herring are common [27,28]. In contrast, species composition north of the Front is typical of high latitudes, with more Arctic species [28,29], such as the large stocks of polar cod (*Boreogadus saida*). These are generally found in the northern and ice-covered portion of the Barents Sea [25,30,31]. However, some boreal species of zooplankton and fish can be advected with the inflow of Atlantic Water across the Polar Front and up to the Arctic Ocean [27,32].

The two most abundant forage fish species in the Barents Sea, capelin and polar cod, are widespread and migrate seasonally across vast distances, from nearshore spawning grounds to offshore summer feeding areas [17,24,31,33]. Both species play a critical role in the transfer of energy from primary consumers to higher trophic levels, including most of the species of commercial importance in the region [17,29,34]. In spring, the Polar Front can be a barrier to capelin northward migration, while not impeding the movement of other species such as polar cod [35]. Seasonal impacts on species movement across the Polar Front have not been studied in detail, and the influence of the Front on the migrations and life histories of pelagic species in the Barents Sea is unclear.

The goal of this study is to assess how hydrographic changes associated with the Barents Sea Polar Front alter the spatial distribution and biomass of fish and macrozooplankton in spring, summer, and winter. Despite the Barents Sea being one of the most well studied Arctic ecosystems [36–38], there is a current lack in high resolution observational data and in situ winter measurements [30,39]. By describing the fine-scale vertical, geographic, and temporal distribution of pelagic organisms across the Polar Front, we aim to expand the current understanding of pelagic ecology in the region,fill some of those knowledge gaps as well as improve fisheries management efforts. Understanding the abiotic and biotic influence on fish populations is a cornerstone in the framework of simulation modeling used in fisheries management [40–43]. Better insight and consideration of the fine scale spatiotemporal distribution of fishes can also guide sampling efforts and resource allocation during monitoring efforts [42], as well as in establishing marine protected areas [43].

## Materials and methods

### Study design

**Fig 1:**
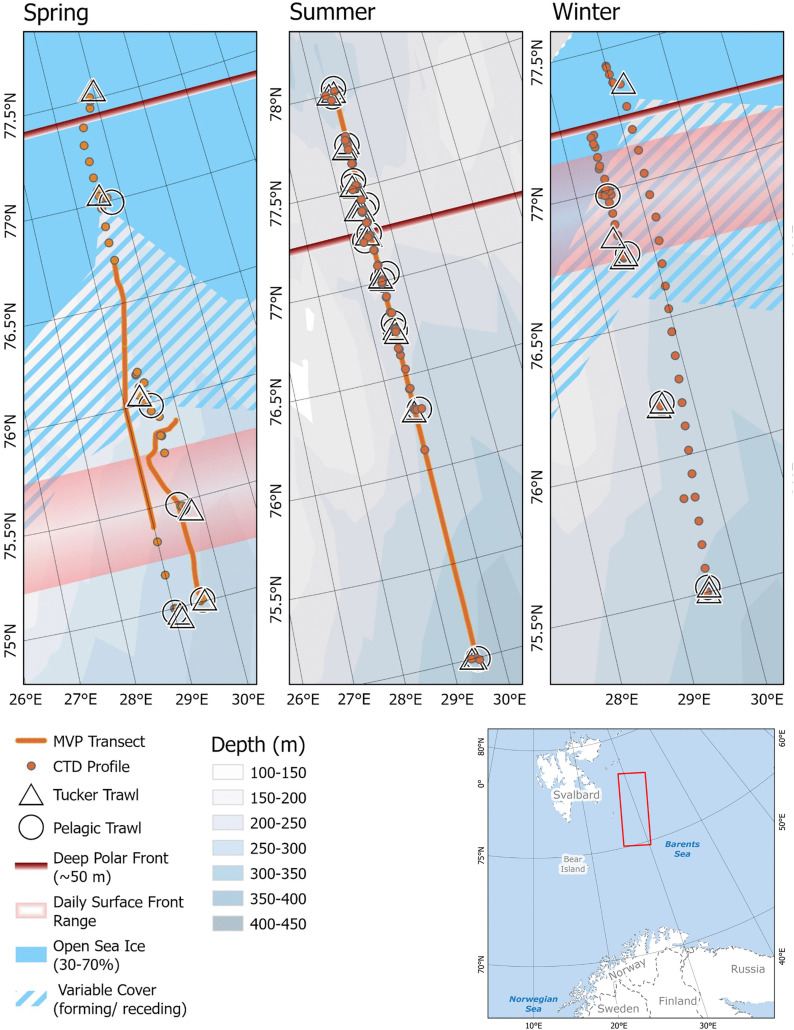
Study area during each seasonal survey including the location of the Polar Front, CTD profiles, MVP transects, and trawls. Basemap obtained from ArcGIS online data, sourced from Esri, GEBCO, NOAA, National Geographic, DeLorme, HERE, Geonames.org, and other contributors.

Data were collected during three scientific cruises (May 2022, August 2023 and January 2024) in the western Barents Sea aboard the R/V *Helmer Hanssen*. Surveys were comprised of stations along a transect following a consistent longitudinal path, roughly 29.5 ^o^E (Fig 1). This location was chosen for the stability of the Front across seasons as well as to avoid any confounding effects from any shallow banks. In spring and winter, the northern extents of the transects reached the seasonal sea ice edge and the Polar Front. In summer, the Polar Front was crossed and the transect continued to 78 ^o^N. In spring and summer sampling stations were chosen after an exploratory transect, using a Moving Vessel Profiler (MVP) and a CTD, to collect hydrographic information and map water mass changes as well as the location of the Polar Front. As environmental conditions did not permit the use of an MVP in winter, an exploratory transect to map hydrography was completed using a rosette mounted CTD. This enabled us to select sampling sites that capture variations in temperature and salinity. At each station sampled, environmental data collection was repeated, and biological data were collected.

### Hydrographic data collection and analysis

Hydrographic data used in analysis was compiled from data collected by three distinct CTD instruments deployed separately throughout the study. At each station, two CTD casts were completed, one with a Sea-Bird SBE 911 plus and a second with a Sea-Bird SBE 19 plus V2 CTD. In spring and summer, an AML Oceanographic CTD, mounted on a Moving Vessel Profiler (MVP), was also used for dedicated high resolution hydrographic transects. The Sea-Bird SBE 911 plus CTD was laboratory calibrated routinely and maintained by UiT, The Arctic University of Norway. Calibration of the Sea-Bird SBE 19 plus V2 CTD and the AML Oceanographic MVP mounted CTD were completed after the conclusion of the three scientific cruises. Data collected during the spring survey were calibrated by measuring the offset of measured temperatures and salinity between the laboratory calibrated CTD (Sea-Bird SBE 911 plus) and the Sea-Bird SBE 19 plus V2 CTD at stable water layers, from profiles in temporal and geographic proximity. The MVP mounted AML CTD was calibrated in the same manner against the Sea-Bird SBE 19 plus V2 CTD. Winter and summer calibrations were completed similarly with the Sea-Bird SBE 19 plus V2 CTD, and the AML CTD from the summer data collection period, calibrated against the laboratory calibrated CTD (Sea-Bird SBE 911 plus).

Calibrated temperature and salinity values from all CTD profiles completed during the survey were used to identify prevalent water masses of the area using criteria described in Sundfjord et al.[44] (Table 1). The water column Brunt–Väisälä frequency (N^2^) was also calculated to measure water column stability and stratification during each season. Data were binned using the thresholds of water stability highlighted in Mojica et al. [45](S3 Table). All calculations were made using the python package GSW-C, Gibbs SeaWater (GSW) Oceanographic Toolbox of TEOS-10 [46]. Interpolated plots were created for both water mass classification and water stability.

**Table 1:** Water mass classification used in study. The limits of the absolute salinity, conservative temperature and density used to classify each water mass are denoted. Classification values are from Sundfjord et al., 2020.

Water mass	Absolute salinity SA	Conservative temperature Θ	Density σθ
Polar water		<= 0 °C	<= 27.97 kg/m^3^
Warm Polar water	< 35.06 g /kg	>0 °C	
Atlantic water	>=35.06 g /kg	>2 °C	
Modified Atlantic water	>=35.06 g/ kg	>0 °C and <=2 °C	
Intermediate Water		>-1.1 °C and <=0 °C	
Cold Barents Sea Dense Water		<=−1.1 °C	>27.97 kg/m^3^

The location of the Polar Front was defined by the strongest temperature gradient change. In seasons with ice cover, the Polar Front was divided into a separate surface front and a deep-water subsurface front (>50 m). In the summer, a distinct surface thermal front was not discernible as the surface water was continuously warm (5.1^o^C - 7.9 °C; NOAA OI SST V2 High Resolution Dataset) with a slight gradual latitudinal temperature gradient. Data used to define the surface front were obtained from NOAA OI SST V2 High Resolution Dataset, NOAA PSL [47]. The dataset incorporates satellite and in situ measurements to create an interpolated grid, 0.25^o^ x 0.25^o^ cells, of daily temperature estimates, covering the entirety of the study area. Temperatures measured in-situ during the surveys were used to define the deep-water, subsurface polar front. To define the location of the subsurface front, measured temperatures from 45 m to 55 m were averaged and interpolated across the latitudinal range of the transect with a resolution of 0.01^o^, and the location of the largest change was identified as the deep-water polar front. The depth used to define the subsurface front was chosen based on the calculated mixed water depth and the temperature and salinity profiles, as that depth was below surface mixing and the location where both temperature and salinity began stabilizing. The daily location of the sea-ice edge, for both open ice (30%−70% sea ice concentration) and closed ice (>70% sea ice concentration), was obtained from the Copernicus Climate Change Service (C3S), Climate Data Store (CDS) [48] and used in the analysis of the spring and winter data.

### Acoustic data analysis

During each survey, a continuous acoustic recording was produced using the keel-mounted EK60 split-beam echosounder at 18, 38, and 120 kHz. Ping rate was set to 1 second and pulse length was set to 1,024 µs. The echosounder was calibrated annually following the standard sphere method [49]. Acoustic data were cleaned and analyzed using Echoview 15. Salinity, temperature, and sound velocity measurements made by the dual station Sea-Bird CTD casts and the MVP mounted AML CTD were used in correcting for in situ sound velocity and absorption. Values used in corrections were obtained from CTD casts closest both geographically and temporally to the acoustic data. In summer and winter, sound velocity was calculated in real time by the Sea-Bird CTD and the MVP mounted AML CTD [50]. In spring, sound velocity was calculated using the python package GSW-C, a Gibbs SeaWater (GSW) Oceanographic Toolbox of TEOS-10 [46]. Sound velocity, salinity and temperature measurements from all CTD casts, geographically and temporally close to acoustic data recordings, were averaged to calculate the absorption coefficient at each frequency [51]. All three frequencies were resampled to match ping times to the 38 kHz transducer prior to cleaning the data. One and a half meters above the recorded bottom were removed to exclude the bottom dead zone [52], manual adjustments were made where bottom detection erroneously identified portions of the sea floor. Water surface noise was removed using Echoview’s near-field depth estimation operand. We applied the operand, which estimates the transducer’s on-axis range of the near field (Fresnel zone) and removed noise further by lowering the line 2 meters. This process on average removed the top 12 m of the water column. When needed, the surface excluding line was manually adjusted to remove increased surface noise, e.g., due to air bubbles, or ice breaking. Background noise and signals with a signal to noise ratio < 10 dB were removed using Echoview’s Background noise removal algorithm [53]. Impulse noise removal was then used to remove electrical noise from the various deployed instruments [54]. Settings for the smoothing and context window parameters of the impulse noise removal were either 3 samples by 3 pings or 5 samples by 5 pings depending on the daily level of noise, making sure the least amount of data were lost. Data were then resampled again and smoothed over a 5 samples by 5 pings window. A minimum volume backscattering (S_v_) threshold of −80 dB re 1m^-1^was used on the acoustic data to remove weak signals assumed not to originate from macrozooplankton or nekton.

Clean acoustic data were gridded into a high-resolution 1 m by 0.1 nmi (0.19 km) integration cells and a secondary lower resolution grid of 3 m by 0.25 nmi (0.19 km) integration cells. The high-resolution grid was used for integrating acoustic data for the red, green and blue (RGB) color composite images as this cell size ensured vertical bins could be easily associated with vertical profiles of water parameters and capture small scale changes in the pelagic signal across the Polar Front but not overwhelm computer processing capabilities. While the lower resolution grid was used to integrate data for statistical analysis, to avoid the oversampling of acoustic data.

Acoustic data were then classified as either fish or macrozooplankton. The difference in echo-integrated mean volume backscattering (MVBS in dB re 1 m^-1^) between integrated cells at 38 kHz and 120 kHz (ΔMVBS_120−38_) was used to classify cells with the presence of pelagic fishes and those with signal from macrozooplankton. A ΔMVBS_120−38_ of 5 dB was used as the upper limit of signal from fish [55,56] and a ΔMVBS_120−38_ over 10 dB was used to select signal from macrozooplankton [57]. Due to the increased attenuation of sound with depth and the decreasing signal-to-noise ratio, observational range decreases with increasing transducer frequency [57]. Multifrequency comparison was thus limited to the first 150 m due to the maximum observational range of the 120 kHz frequency. Signal detected at 38 kHz below 150 m was assumed to be fish based on the wavelength of the 38 kHz signal (39 mm). The nautical area scattering coefficients (NASC m^2^ nm^-2^) for selected fish and zooplankton cells was then integrated over the water column with a 0.25 nmi (.05 km) spatial resolution and used as an index of abundance [2,58,59].

Red, green and blue (RGB) color composite images were created using exported acoustic data, to visualize pelagic fish and macrozooplankton distribution across the transects [60,61]. Each recorded frequency was assigned a color (red = 18 kHz, green = 38 kHz, and blue = 120 kHz) and all three frequencies were visualized on a single image. Due to the unique frequency response of pelagic species, the measured signal at each frequency is typically associated with the presence of a distinct group of organisms [62]. The minimum animal size detectable at each frequency is limited by the wavelength of emitted signal, thus the minimum sizes detected with those frequencies used were 81 mm, 39 mm and 12 mm for the 18 kHz, 38 kHz, and 120 kHz respectively. The 18 kHz and 38 kHz therefore captured the presence of gas bladder fishes while the 120 kHz captured larval fish and those macrozooplankton over 12 mm in length or dense patches of larger mesozooplankton, including krill, amphipods, pteropods, and copepods [62]. A composite image created from acoustic data can therefore highlight predominant frequencies and the corresponding species as well as those areas where species presence overlaps (mixed color: white, pink, teal, or yellow). In preparation of the data, care was taken to use as continuous of a transect as possible and not include ship back-tracking or circling in one geographic area by removing those portions. The mean volume backscatter (MVBS dB re 1 m ^−1^) of each cell was transformed to a color index value on a scale of 256 using the following equation [60]:


$$\;Color\;index\;=(\frac{\text{2}\text{5}\text{5}}{\;(Maximum\;MVBS-Minimum\;MVBS))}\times \;({MVBS}_{(fr)\;}-\;Minimum\;MVBS)\;$$

Where MVBS_(fr)_ is the MVBS of the cell being transformed and the minimum and maximums values of MVBS in the entire transect were used as a reference for the rescaling. The rescaled values of each frequency were assigned a color (red = 18 kHz, green = 38 kHz, and blue = 120 kHz) and used as inputs to build composite images of each season’s transect across the Front. Images were constructed with the Matplotlib package in Python [63]. Data were brightened and gamma normalized (gamma = 2) for better visualization.

### Pelagic sampling

Macrozooplankton were sampled using a Tucker trawl. In total 47 tows were completed over the three seasons (Fig 1, S1 Table). The single, rectangular, fixed frame net had an opening of 1 m^2^ with a mesh size of 1000 μm. Tow depth was determined based on the sound-scattering layers identified on the live echogram from the keel mounted EK60 echosounder. Each deployment of the net included 10−16 minutes at the target depth while cruising at 2 knots (3.7 km h^-1^). Tow times were calculated based on when the net was submerged and the boat was moving at towing speeds. During the spring and winter surveys (May 2022 and January 2024), Tucker trawl sampling was limited to where sea ice was sparse and allowed for trawling. Organisms caught were identified to the highest taxonomic resolution possible onboard and each taxonomic group was enumerated. Species caught in large quantities (i.e., Chaetognaths, Euphausiids) were subsampled based on volumetric splitting and the total count was calculated based on the number of individuals in the subsample. All species too small to identify and separate with the naked eye were grouped together (mixed mesozooplankton, MMZ). This grouping of MMZ was primarily composed of individuals from the class Copepoda, but no further distinction was made for the purpose of this analysis, as the Tucker trawl is unable to capture a true representation of the entire mesozooplankton community. Biomass of this group was included in further analysis due to it being detectable by the echosounder. Separated specimens were rinsed with freshwater and subsequently dried on pre-weighed dishes at 60^o^ C for a minimum of 48 hours to ensure complete desiccation and constant mass [64]. Dry weights were measured once on land.

Fish were sampled when ice permitted trawling and in proximity to Tucker trawls. A Harstad pelagic trawl with an opening of approximately 80 m^2^ and a cod end with a mesh size of 5.5 mm was towed at roughly 3 knots (5.6 km h^-1^) for 15–30 min. As with the Tucker trawl, tow depth varied by station and was determined based on the predominant sound-scattering layer. Twenty-four trawls were completed over the three seasons (Fig 1, S1 Table). All organisms brought onboard were identified to the highest taxonomic resolution possible. The total weight and number of individuals of each taxonomic grouping were recorded. A subsample of 20–25 individuals of each species of fish caught was also individually weighed and measured.

### Ethics statement

This study was carried out in accordance with the Norwegian animal welfare authorities and followed the strict regulations regarding health, environment and safety enforced at UiT (The Arctic University of Norway). Trawling was carried out with permission from the Norwegian Directorate of Fisheries. At present, it is not necessary to acquire special permissions for the scientific sampling of wild fishes from an animal welfare or equivalent ethics committee in Norway. All efforts were made to reduce suffering and stress of fishes by quick removal from nets, but due to the vessel net configuration and layout being that of a commercial fishing vessel as well as environmental conditions, fishes were deceased shortly after landing and did not require additional euthanasia methods prior to processing.

### Statistical analysis

A correlation analysis using Kendall Tau correlation coefficient was run using the calculated Nautical Area Scattering Coefficient (NASC) values for macrozooplankton and fish. Fish NASC was separated by depth so that Fish NASC from above 150 m (shallow water fish) and below 150 m (deep water fish) were analyzed separately. The two depth classes likely represent different age classes of the species encountered, as many species are depth stratified by age with older individuals found in deeper waters [65,66]. The level of correlation of NASC among all three groups (macrozooplankton, deep water fish, and shallow water fish) as well as to latitude, distance to the Front (km), salinity and temperature were calculated. In situ water temperature and salinity used in analysis correspond to the depth division in the acoustic data and correlations were completed with measurements that reflect water properties within the different depth strata. Correlation statistical significance (p values) were adjusted for false positives using the methods described in Benjamini and Hochberg 1995 [67]. Analysis was completed using the R packages Performance Analytics and Stats.

Random forest regressors were used to measure abiotic and biotic variables importance on fish and macrozooplankton biomass (NASC) across the Polar Front during each season. and to identify drivers of fish and macrozooplankton distribution of across the front. Random forest regression is a decision tree based machine learning approach used to predict or classify data [68] and is well suited for ecological studies because it is able to handle complex non-linear relationships among many variables, in a spatially independent manner [68–71]. Effective implantation of random forest regressors involves accurate hyperparameterization of the model. While regression predictions are ultimately not affected by collinearity of explanatory variables [70], variable importance is affected by collinearity [72]. Prior to tuning each random forest regression used in this study, variance inflation factors (VIF) were used to identify collinearity among explanatory variables each season. For each season, the best fitting random forest regressor involved a unique group of biotic and abiotic explanatory variables, and all models included biological, geographic, and physical environment variables. The final variables chosen were based on both ecological knowledge as well as avoiding high collinearity (>10) among factors. Multiple combinations of independent explanatory variables were tested and the combination of variables that provided the best model output (highest R^2^) across the different testing variables was chosen for each season. Hyperparametrization was done on each of the six random forest regressions separately using a grid search of a range of parameters. All model training and validation were done in ArcPro. In all cases final regressors were built and tested with a 7:3 ratio (70% training data, 30% testing data). Once the optimal model was identified it was tested on excluded data five times to measure accuracy. Final model parameters and grid search ranges for each of the six of the models ran are presented in detail in S5 Table.

Trawl catches were standardized by the volume of water trawled (net opening area multiplied by distance towed), and all analyses were conducted on these standardized values. Standardized species counts were used to calculate the Shannon-Wiener and Simpson’s diversity indices for both macrozooplankton and fish caught. Diversity indices were calculated for macrozooplankton without the MMZ portion of the catch, because the MMZ grouping was not enumerated. All other macrozooplankton community comparisons across seasons and water masses were based on the dry weights of taxonomic groups and included the biomass of the MMZ assemblage group. Biocore/scikit-bio Python package [73] was used for calculation of diversity indices. Fish and macrozooplankton community comparisons across seasons and water masses were completed using ANOSIM and visualized through non-metric multidimensional scaling (NMDS). Community changes across seasons and water masses were further analyzed by comparing ordinated means across seasons and water masses using PERMANOVA as well as by measuring the statistical difference of site dispersion using PERMDISP. All community comparisons were completed using the R package, Vegan [74]. All three calculations were run as a multifactor test for both season and water mass. Square root transformed relative abundance was used when calculating distance matrices used in the multivariate analyses due to catches of both macrozooplankton and fishes being dominated by a few species. Kendall’s Tau correlation statistics were calculated for both diversity and species abundance to try to identify drivers of distribution.

## Results

### Distribution of acoustic backscatter across water masses

**Fig 2:**
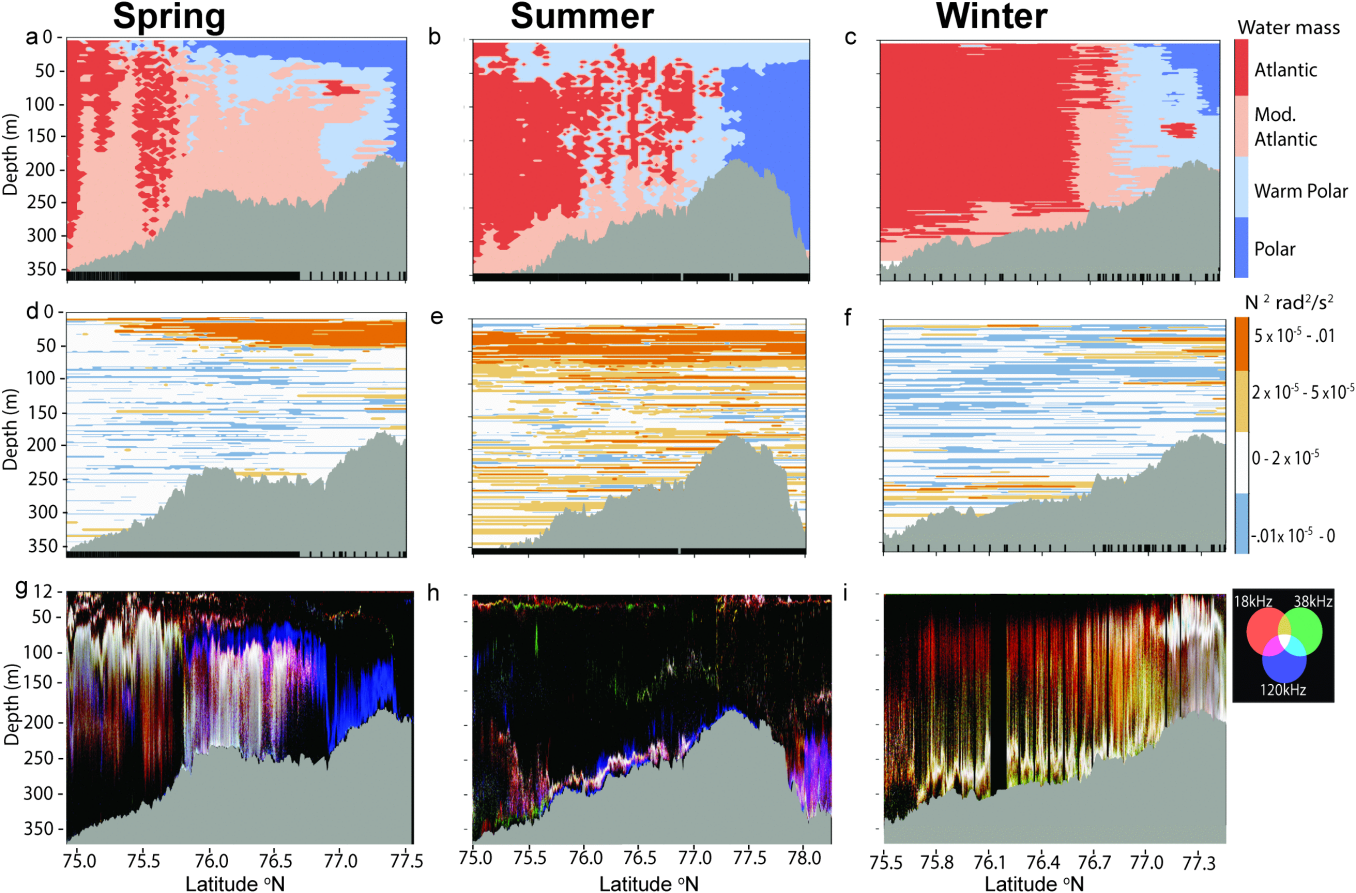
a-i. Water mass classification (a-c), water stability (d-f), and pelagic signal (g-i) in each season. Black bars at bottom of water classification and stability plots indicate locations of water parameter profiles. Note: horizontal (latitude) axis extends further north in summer than the other seasons.

Temperature and salinity across the Barents Sea Polar Front varied seasonally. In seasons when sea ice was present, shallow Polar water created a distinct surface front. This was most pronounced in spring where the Polar Front was shaped as an upside-down L with a vertical wall of Polar Water at around 77.37 ^o^N and a long arm of surface Polar waters in the upper 50 m of the water column extending southward to between 75.21^o^ N and 75.73 ^o^N, depending on the day (Fig 2a, S2a and S2b Fig). In situ measurements ranged from 3.2 °C south of the Polar Front to −1.8 °C north of the Front, and satellite-derived average sea-surface temperatures varied from 2.9 °C to −1.8 °C. In winter, the surface front was less pronounced than in spring and was found 11−55 km south of the deep-water front, depending on day: The daily surface front ranged from 76.7 ^o^N to 77.1 ^o^N, and the deep-water front was at roughly 77.20 ^o^N. Satellite-derived average temperatures varied from 3.0 °C to −1.5 °C and measured temperature varied from 3.2 °C south of the Polar Front to −1.8 °C north of the Front. During the summer survey, sea ice was absent, a temperature surface front was not distinguishable as surface waters were highly stratified and a layer of warm water (5.1^o^ - 7.9 °C, satellite derived) was present across the entire transect without a clear latitudinal gradient. At depth, temperature varied from 9.3 °C south of the Polar Front to −1.8 °C north of the Front. Summer was the only season where Cold Barents Sea Dense Water (CBSDW, Table 1) was encountered. This small pocket of cold and dense water was observed at around 100 m depth north of the Polar Front, and although it was sampled with all three instruments deployed for hydrographic measurements and some nets, it was too small to be captured by the interpolated water mass classification plots (Fig 2b). The deep-water Front in summer appeared as a vertical wall at around 77.22 ^o^N (Fig 2a-2c) starting from a depth of 50 m.

Acoustic backscatter of both zooplankton and fish appeared to correspond and vary with changes in water masses in all seasons (Fig 2g-2i). Atlantic waters in both spring and summer had higher recorded acoustic backscatter than Polar waters, while in winter, higher acoustic backscatter was recorded in Polar waters. Spring was unique in that a dominant band from the 120 kHz, without any mixing from the lower frequencies, was visible from around 76.8 ^o^N to the subsurface Front at 77.37 ^o^N. This band, of what is likely macrozooplankton and some mesozooplankton clusters, appeared to be present in Warm Polar waters and Polar waters. The sound scattering layer in spring, at 18 and 38 kHz, appeared to be confined to Atlantic waters and even increased in depth to stay within the water mass. In summer, the water column was surprisingly empty in comparison to winter and spring, with most of the fish and macrozooplankton biomass concentrated close to the sea floor. This concentrated deeper layer, likely a mixed-species assemblage (strong backscatter from all three frequencies), was also present in winter. In winter, backscatter from what was likely fishes (18 kHz and 38 kHz only) was visible throughout the water column for the entire transect, increasing to a much higher concentration in Polar waters and under sea ice. Winter was the only month when such a strong signal was recorded in proximity to the subsurface front.

### Pelagic fish

#### Fish abundance.

**Fig 3:**
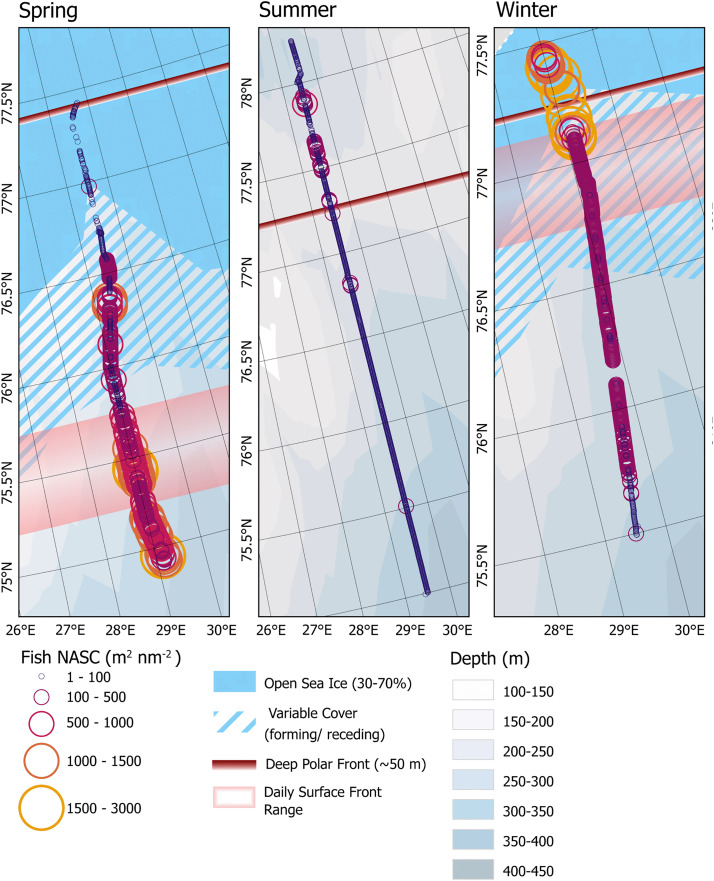
Pelagic fish NASC. Identified fish NASC from the top 150 m of water. Symbols are proportional to the calculated NASC.

Average fish abundance and distribution estimated from acoustic transects (based on NASC re 0.1 nmi^-1^) differed greatly over the three seasons. Geographic distribution patterns of fishes across the transect in shallow-water (<150 m, obtained from ΔMVBS_120–38_) differed slightly from deep-water (>150 m, obtained from 38kHz NASC) values in each season, with the difference being most notable in summer where fish biomass appeared absent from shallow-water and primarily concentrated directly above the seabed (Fig 2h). Fish NASC in the upper 150 m was derived differently than the NASC of fishes in deeper waters, so no direct comparison of values between the two depth classes was made and any comparison between shallow and deep-water fish abundance was limited to highlighting the patterns in NASC magnitude across the Front. Overall fish abundance was highest in winter, both in the upper water layer and in deep-water. During that season, average shallow water NASC was estimated at 1591.0 m^2^ nm^-2^ (sd = 4542.5, range = 0.0–35895.4) and average deep water NASC was estimated at 145.4 m^2^ nm^-2^ (sd = 122.2, range = 0.0–904.4). These high estimates were largely driven by the very strong backscatter at the Front and the ice edge (Fig 3). In spring, abundance was highest in the southern portion of the transect, below 75.56 ^o^N, and decreased northwards towards the subsurface front and the ice edge. The spring average NASC in shallow water was 266.4 m^2^ nm^-2^ (sd = 356.0, range = 0.0–2148.6 m^2^ nm^-2^) and was 50.5 m^2^ nm^-2^ (sd = 56.2, range = 0.0–333.5. m^2^ nm^-2^) in deep water. In summer, shallow water NASC did not vary much across the transect and was low compared to both spring and winter (25.6 m^2^ nm^-2^, sd = 60.0, range = 0.0–885.2 m^2^ nm^-2^). Average deep water NASC in summer was comparable to that of spring with 34.6 m^2^ nm^-2^ (sd = 63.73, range = 0.1–904.4 m^2^ nm^-2^). Average deep water NASC in summer appeared to decrease slightly near the subsurface front, with the highest NASC measured in the northernmost portion of the transect (S3 Fig).

The strongest significant correlation between pelagic fish abundance (NASC re 0.1 nmi^-1^) and the Polar Front was observed in spring. Pelagic fish abundance, both in shallow and deep water, were significantly negatively correlated with distance to the daily surface front (Τ = −0.67, adj.p= < .0001, Τ = −0.39, adj. p= < .00001, respectively), and strongly positively correlated with the distance from the ice edge and deep Front (Table 2). Summer had weak and non-significant correlations between abundance of fish in shallow water and hydrographic parameters at the front. Deep fish NASC showed significant positive correlation to the distance from the front, and negative correlation to latitude. Winter was the only season where fish NASC in both the deep and shallow waters were negatively correlated to distance from the subsurface deep front.

**Table 2:** Kendal Tau correlation coefficient table of water column NASC and hydrographic parameters at the Front. Color indicates strength of correlation (darker color = stronger correlation) with red showing a significantly positive correlation and blue showing a significantly negative correlation. P value has been adjusted using the methods from Benjamini and Hochberg, 1995 for false positive detection in multiple comparisons [67]. Bolded terms are significant.

		*Corr. Coef.*	*p Adjusted*	*Corr. Coef.*	*p Adjusted*	*Corr. Coef.*	*p Adjusted*
***Shallow water fish***	*Distance from closed ice*	**0.70**	**p < 0.001**			**−0.55**	**p < 0.001**
	*Distance from open ice*	**0.68**	**p < 0.001**			**−0.55**	**p < 0.001**
	*Distance from Surface front*	**−0.67**	**p < 0.001**			**−0.37**	**p < 0.001**
	*Distance from deep front*	**0.69**	**p < 0.001**	0.00	p > 0.05	**−0.55**	**p < 0.001**
	*Latitude*	**−0.70**	**p < 0.001**	−0.01	p > 0.05	**0.55**	**p < 0.001**
	*Sal. middle*	**0.65**	**p < 0.001**	0.00	p > 0.05	**−0.49**	**p < 0.001**
	*Sal. bottom*	**0.30**	**p < 0.001**	0.00	p > 0.05	**−0.52**	**p < 0.001**
	*Temp. surface*	**0.63**	**p < 0.001**	0.03	p > 0.05	**−0.56**	**p < 0.001**
	*Temp. middle*	**0.64**	**p < 0.001**	0.00	p > 0.05	**−0.55**	**p < 0.001**
	*Temp. bottom*	**0.64**	**p < 0.001**	−0.01	p > 0.05	**−0.50**	**p < 0.001**
	*Macrozooplankton NASC*	**−0.39**	**p < 0.001**	−0.01	p > 0.05	−0.03	p > 0.05
	*Deep water fish NASC*	**0.46**	**p < 0.001**	−0.02	p > 0.05	**0.65**	**p < 0.001**
***Deep water fish***	*Distance from closed ice*	**0.45**	**p < 0.001**			**−0.30**	**p < 0.001**
	*Distance from open ice*	**0.42**	**p < 0.001**			**−0.26**	**p < 0.001**
	*Distance from Surface front*	**−0.39**	**p < 0.001**			**−0.15**	**p < 0.001**
	*Distance from deep front*	**0.44**	**p < 0.001**	**0.39**	**p < 0.001**	**−0.24**	**p < 0.001**
	*Latitude*	**−0.45**	**p < 0.001**	**−0.29**	**p < 0.001**	**0.30**	**p < 0.001**
	*Sal. middle*	**0.39**	**p < 0.001**	**0.26**	**p < 0.001**	**−0.27**	**p < 0.001**
	*Sal. bottom*	−0.04	p > 0.05	**0.30**	**p < 0.001**	**−0.30**	**p < 0.001**
	*Temp. surface*	**0.33**	**p < 0.001**	**0.32**	**p < 0.001**	**−0.29**	**p < 0.001**
	*Temp. middle*	**0.39**	**p < 0.001**	**0.25**	**p < 0.001**	**−0.30**	**p < 0.001**
	*Temp. bottom*	**0.40**	**p < 0.001**	**0.30**	**p < 0.001**	**−0.28**	**p < 0.001**
	*Macrozooplankton NASC*	**−0.09**	**p < 0.05**	−0.05	p > 0.05	**0.11**	**p < 0.05**
	*Shallow water fish NASC*	**0.46**	**p < 0.001**	−0.02	p > 0.05	**0.65**	**p < 0.001**
***Macrozooplankton***	*Distance from closed ice*	**−0.50**	**p < 0.001**			**0.18**	**p < 0.001**
	*Distance from open ice*	**−0.47**	**p < 0.001**			**0.18**	**p < 0.001**
	*Distance from Surface front*	**0.46**	**p < 0.001**			**0.15**	**p < 0.001**
	*Distance from deep front*	**−0.51**	**p < 0.001**	0.08	p > 0.05	**0.18**	**p < 0.001**
	*Latitude*	**0.50**	**p < 0.001**	−0.08	p > 0.05	**−0.18**	**p < 0.001**
	*Sal. middle*	**−0.47**	**p < 0.001**	**0.09**	**p < 0.05**	**0.19**	**p < 0.001**
	*Sal. bottom*	**−0.40**	**p < 0.001**	0.08	p > 0.05	**0.19**	**p < 0.001**
	*Temp. surface*	**−0.50**	**p < 0.001**	**0.13**	**p < 0.001**	**0.18**	**p < 0.001**
	*Temp. middle*	**−0.49**	**p < 0.001**	0.08	p > 0.05	**0.21**	**p < 0.001**
	*Temp. bottom*	**−0.49**	**p < 0.001**	**0.09**	**p < 0.05**	**0.22**	**p < 0.001**
	*Deep water fish NASC*	**−0.09**	**p < 0.05**	−0.05	p > 0.05	**0.11**	**p < 0.05**
	*Shallow water fish NASC*	**−0.39**	**p < 0.001**	−0.01	p > 0.05	−0.03	p > 0.05

**Fig 4:**
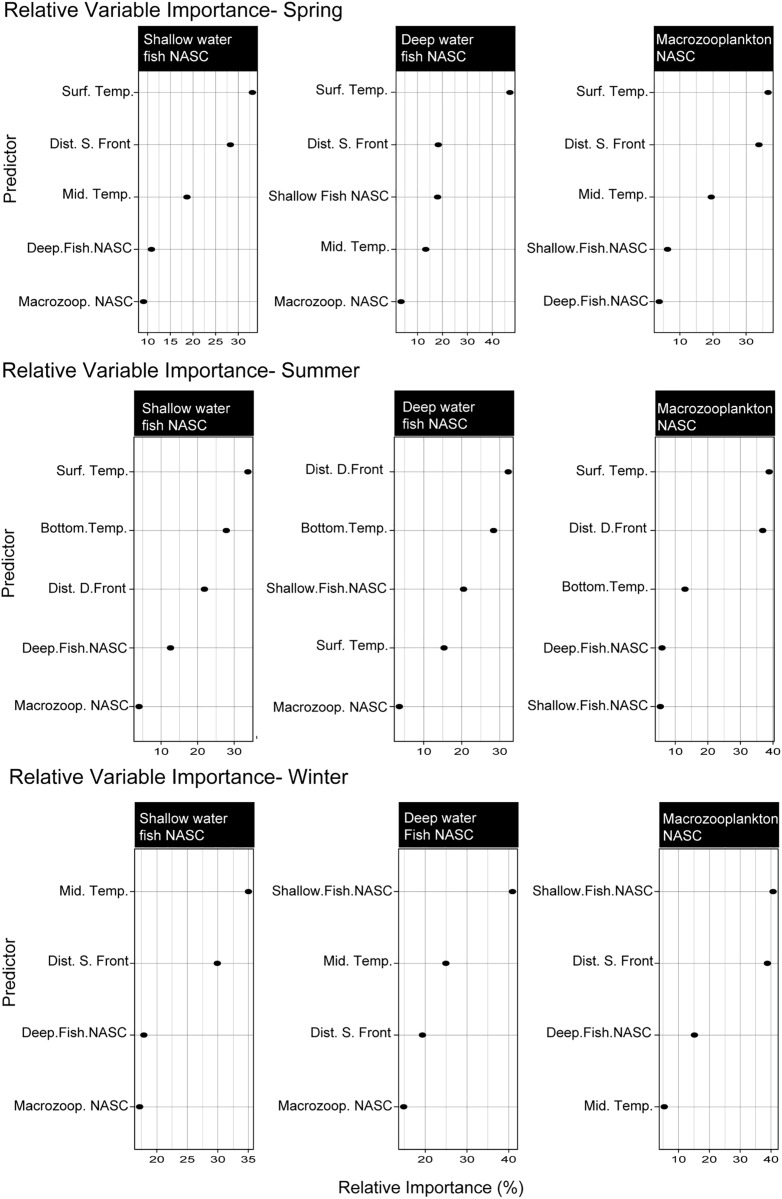
Random forest relative variable importance plots for each season. The significance of the variables was ranked in order of percent importance with the variable of highest importance listed at the top and the lowest at the bottom.

The importance of variables measured by the random forest regression to predict both shallow and deep fish NASC differed seasonally. Temperature, regardless of depth, was found to be one of the most important explanatory variables across all seasons. Geographic position, in relation to the Polar Front, was also found to be of importance and often ranked higher than biological variables. Variable importance in spring predictive models for shallow and deep fish NASC were similar, with the top two most important variables being sea surface temperature (SST) and geographic distance to the surface front. The lowest ranked variable in both models was zooplankton NASC (Fig 4) which explained less than 10% of the variation. In summer, the most influential variable in predicting shallow fish NASC was SST with bottom temperature being second most important. Distance from the deep front and bottom temperatures were the most important explanatory variables for deep water fish NASC. Macrozooplankton NASC was again the least important predictive variable to deep and shallow fish NASC. While abiotic variables were most important in the modeling of fish NASC across the Polar Front in spring and summer. In winter, the most important variable in modeling deep water fish NASC was shallow water fish NASC.

#### Fish catch density.

Average pelagic fish density (standardized, g m^-3^) was highest in spring and winter, both 0.24 g m^-3^ (sd = 0.26, sd = 0.40, respectively), and lowest in summer (0.02 g m^-3^, sd = 0.05). No significant correlation was found between individual fish species density and measured hydrographic parameters or with distance to the Polar Front. The small sample size of pelagic trawls during spring and winter (n = 5 in spring and n = 4 in winter) likely limited the significance of the correlation analysis.

**Fig 5:**
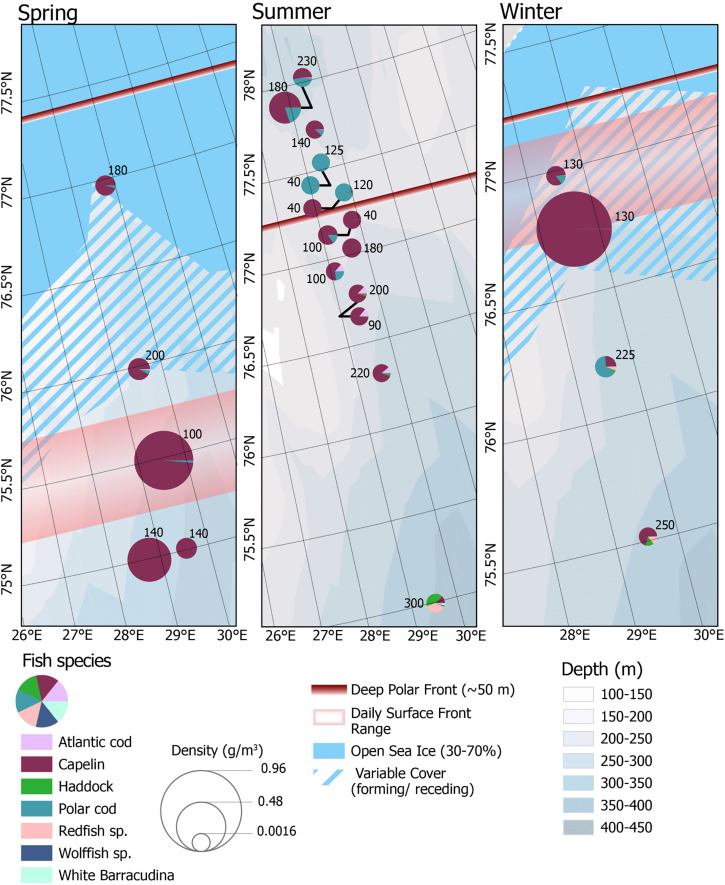
Pelagic fish biomass and relative abundance in net catches. Symbols are proportional to the total density of the catch, and the number indicates the depth of the net deployment. The relative abundance of the seven most abundant fish species is represented by the pie chart symbol. The daily surface front was not recognizable in summer.

During all three seasons, capelin (*Mallotus villosus*) was the most abundant fish caught (Fig 5). In spring the species accounted for over 90% of all fish. In both summer and winter, pelagic trawl catches were more diverse, although in those seasons capelin still accounted for more than half of fish caught in all but a few sites. Young of the year capelin were caught in all three cruises, with the majority captured in winter. Winter was also the only season when several gravid adult capelin were captured. In the summer, a notable large number of capelin were caught in deep Polar water north of the Polar Front, although overall catches had no obvious significant correlation with the location of the Front or any hydrographic parameters.

The second most abundant fish in all seasons was polar cod (*Boreogadus saida*). Density of polar cod was not found to be significantly correlated with latitude, proximity to the Front or ice edge. The species was caught both north and south of the Front. In summer, the species was more abundant north of the Polar Front, with an average of 0.006 fish/m^3^, compared to an average of 0.001 fish/m^3^ south of the front. Polar cod was most abundant in summer, when many young of the year, 7132 individuals (2060 g), were caught. Redfish (*Sebastes* sp*.*) and haddock (*Melanogrammus aeglefinus*) were also caught during all three seasons, but both species were most abundant in winter, with catch averages of 0.0002 and 0.0001 fish/m^3^ for redfish and haddock respectively. Atlantic cod (*Gadus morhua*) were caught at an average catch density of 3 x 10^−5^ fish/m^3^ in winter, 4 x 10^−5^ fish/m^3^ in summer, and none in spring. Although the average catch density of Atlantic cod was lower in winter than in summer, the species was caught in all trawls across the transect in winter, while in summer the species was found in only nine of the 15 trawls. No significant correlation was found between density of any species of pelagic fish and the hydrographic parameters defining the Front or ice edge.

#### Fish diversity.

Fish diversity and species distribution as estimated from trawl catches varied geographically across the Polar Front as well as seasonally, although community composition was not statistically different across seasons. Pelagic fish diversity peaked in winter (average Shannon-Wiener index = 0.60, sd = 0.41) and was the lowest in spring (average Shannon-Wiener index = 0.11, sd = 0.14) (a complete list of biodiversity values for each site is provided in S3 Table). No significant correlations between catch density of any species of fish or net catch diversity and the hydrographic features of the Polar Front were found.

**Fig 6:**
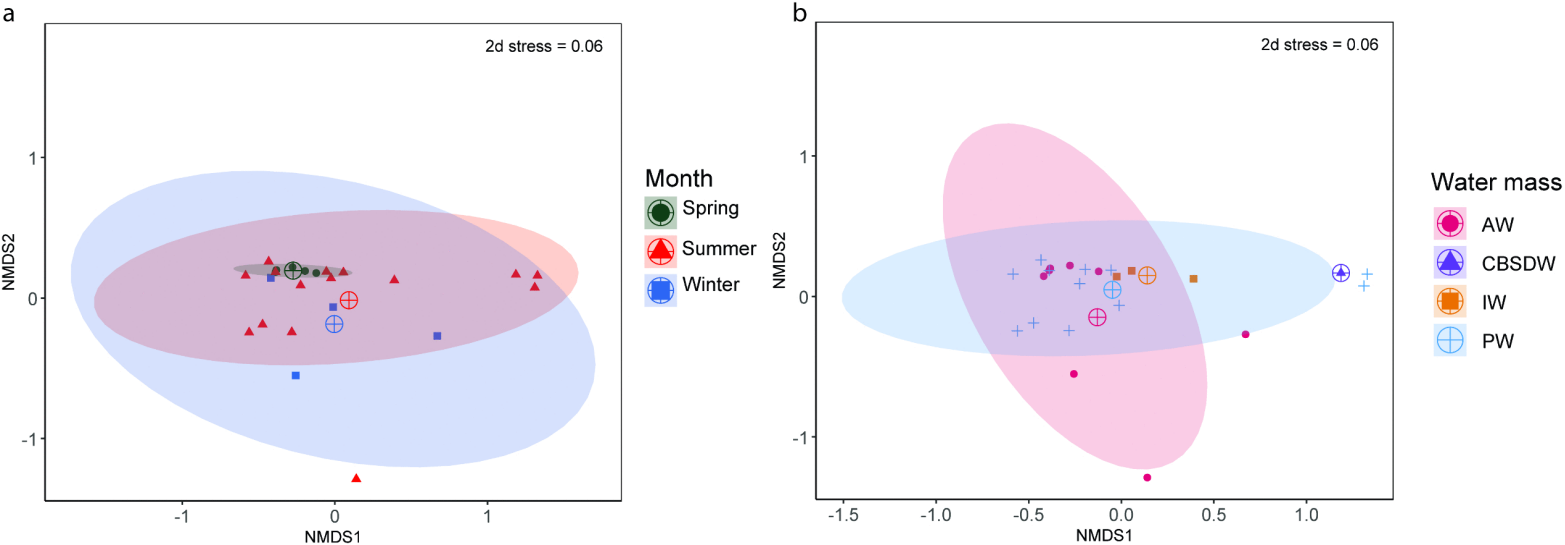
Pelagic fish community comparison visualized through non-metric multidimensional scaling (NMDS, k = 2 dimensions). **a)** Seasonal grouping of fish communities.Stations sampled in spring are indicated in green, those sampled in winter are in blue, and those sampled in summer are in red. **b)** Pelagic fish communities grouped by water mass. Intermediate water in yellow, Atlantic water in red, Polar water in blue, Cold Barents Sea Dense Water in purple. Group centroid is marked by a target symbol.

No statistical difference was found in fish communities across the three sampling seasons or the sampled water masses (ANOSIM: p = 0.80, p = 0.20 respectively) (Fig 6), and no pairwise comparisons across seasons or water masses were found to be significant. Across water mass types, both PERMANOVA and PERMDISP showed no statistical difference among the group centroid value and dispersion distances (p = 0.10, p = 0.20 respectively). When grouping by season, results from the PERMANOVA were not significant (p = 0.20), but results from the PERMDISP were significant (p = 0.01). Catches in spring were tightly clustered around the group’s centroid compared to those of summer and winter. Although square-root transformed data were used in analysis, the large number of capelin and polar cod could be masking some nuanced changes among sites and seasons. Due to only a small number of trawls in each defined water mass, a comparison of community changes across all six water masses was not possible. For fish community analyses, the water masses identified were thus grouped by the originating source: Warm Polar water were grouped with Polar water and modified Atlantic waters were grouped with Atlantic water.

### Macrozooplankton

#### Macrozooplankton abundance.

**Fig 7:**
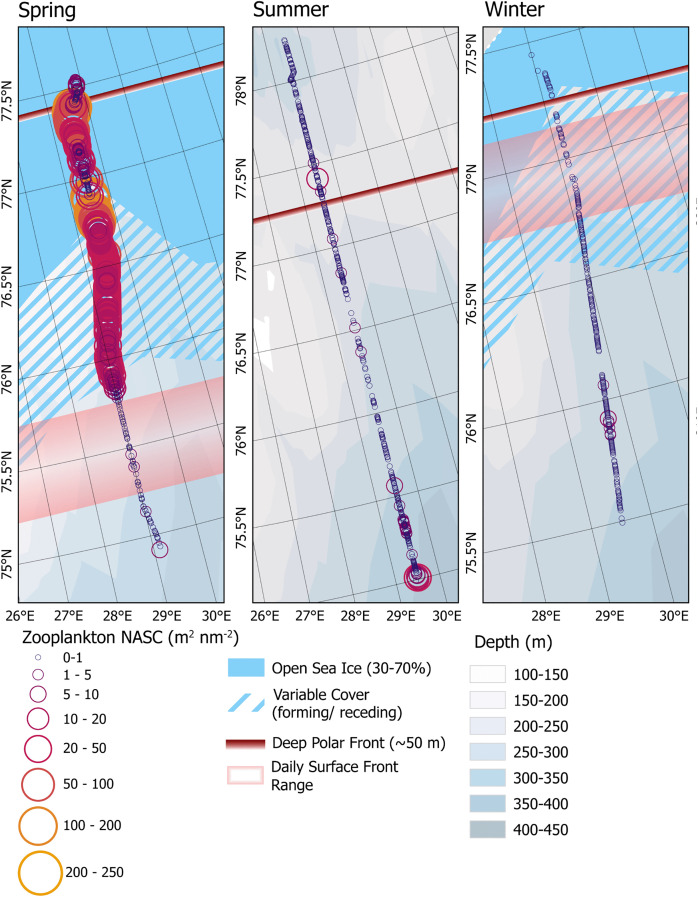
Zooplankton NASC. Identified zooplankton NASC from the top 150 m of water. Symbols are proportional to the calculated NASC.

Acoustically derived abundance estimates of macrozooplankton in the top 150 m of water reflected seasonally distinct latitudinal distributions and relationships to the Polar Front and were different from patterns observed in fish distribution (Fig 7). The highest average macrozooplankton NASC was measured in spring, 24.6 m^2^ nm^-2^ (sd = 31.3). During the spring survey macrozooplankton NASC appeared to be highest north of the surface front and increased towards the deep water front and the ice edge (Fig 7). At the ice edge the macrozooplankton signal weakened and disappeared almost entirely further north. The lowest average NASC was measured in winter (0.15 m^2^ nm^-2^; sd = 0.42).

As with fish abundance, the strongest significant correlations between macrozooplankton NASC and the Polar Front were observed in spring. Macrozooplankton NASC was significantly negatively correlated with distance from sea ice and the deep front but positively correlated to distance from the daily surface front (Table 2). Spring was also the only time where macrozooplankton abundance was positively correlated with latitude (Τ = .062, adj. p < 0.001). In summer, the only significant, although very weak, correlation was between macrozooplankton NASC and salinity (T = .01, p < .005). In winter, correlations between macrozooplankton abundance and hydrographic parameters were weak but all parameters, except for latitude and shallow water fish NASC, were positively correlated with abundance

The importance of each variable in the random forest regressor varied by season. In both spring and summer, SST was the strongest predictive variable in the random forest regression. Distance from the surface front was the second most important variable predicting macrozooplankton NASC in spring, while distance from the subsurface front was the second most important predictive variable in summer. In both spring and summer, the variables with the lowest importance were deep and shallow water fish NASC, with both measurements contributing only slightly to the models. Winter was the only season where a biological variable was ranked as having the highest importance to the model, as shallow fish NASC was ranked as the most influential variable in predicting macrozooplankton NASC. Though, distance from the surface front was the second most influential variable, only slightly lower than shallow fish NASC.

#### Macrozooplankton diversity and density.

Macrozooplankton diversity as determined from the Tucker trawl catches varied geographically and seasonally, but the highest macrozooplankton diversity was measured in the northernmost portions of the transects in all seasons. In winter and summer, there was no significant correlation between macrozooplankton catch density or diversity and the Polar Front hydrographic parameters. In spring, diversity was positively correlated to distance from the surface front (Τ = 0.83, p. adj = 0.03). Macrozooplankton diversity and density were both highest in winter with an average Shannon-Wiener diversity index of 1.16 (sd = 0.07) and an average dry weight of 0.007 g m^-3^ (sd = 0.003). The lowest diversity of macrozooplankton was measured in spring, with an average Shannon-Wiener diversity index of 0.56 (sd = 0.50). Spring macrozooplankton biomass was on average 0.002 g m^-3^ (sd = 0.002), the same as in summer, where the average biomass of 0.002 g m^-3^ (sd = 0.003). Summer diversity was higher than that of spring, with an average Shannon-Wiener diversity index of 1.07 (sd = 0.36) (S4 Table). No significant correlations were found between any taxonomic groups of macrozooplankton communities and the hydrographic features of the Polar Front.

**Fig 8:**
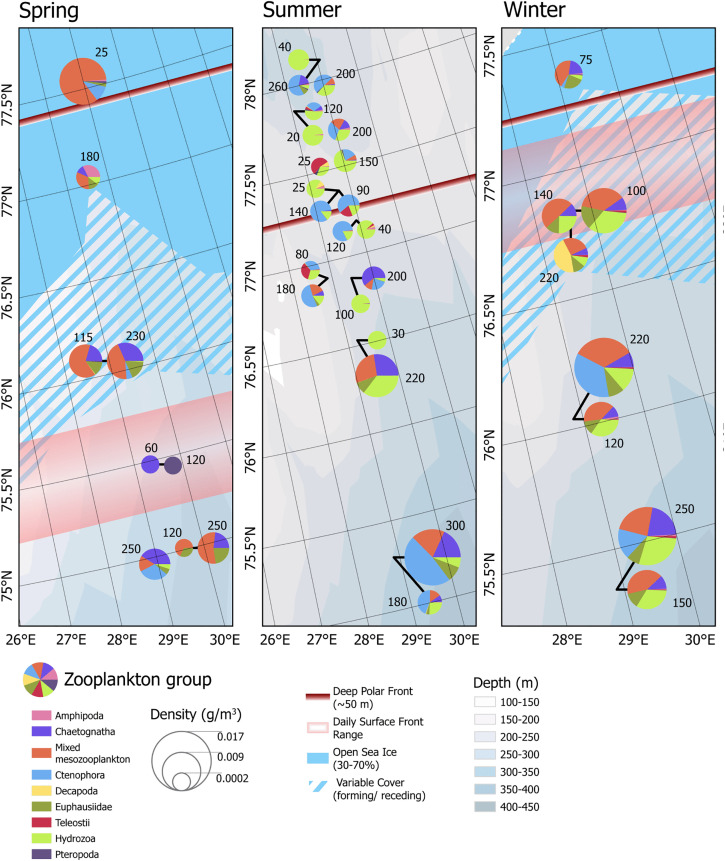
Zooplankton biomass in net catches. Symbols are proportional to the size of the catch, and the numbers indicate the depth of the net deployments.

Macrozooplankton biomass and distribution patterns derived from net catches captured those patterns missed by acoustic data, as nets were able to collect animals past the depth limitation of the frequency used to identify macrozooplankton, as well as capture those organisms that are poor acoustic scatterers. Gelatinous zooplankton are weak sound scatterers [75,76] and are likely poorly detected by the echosounder. Biomass of gelatinous macrozooplankton (hydrozoans and ctenophores) accounted for >20% of macrozooplankton catches in winter, > 40% in summer, and only 9% in spring. The lower proportion of gelatinous zooplankton in spring is likely contributing to the poor agreement of between macrozooplankton NASC and macrozooplankton average catch biomass. In summer the large relative biomass of hydrozoans was primarily composed of the jellyfish *Cyanea capillata* and ctenophore *Beroe cucumis* (Fig 8). In spring, MMZ and euphausiids accounted for a largest percentage of the zooplankton biomass, with an average of 59% of the catch by weight, compared to 49% of the catches in winter and 9% in summer (Fig 8, see also S4 Table). MMZ in all seasons was largely composed of copepods of the genus *Calanus* (pers. observation M. Daase), though no further distinction was made for this analysis.

**Fig 9:**
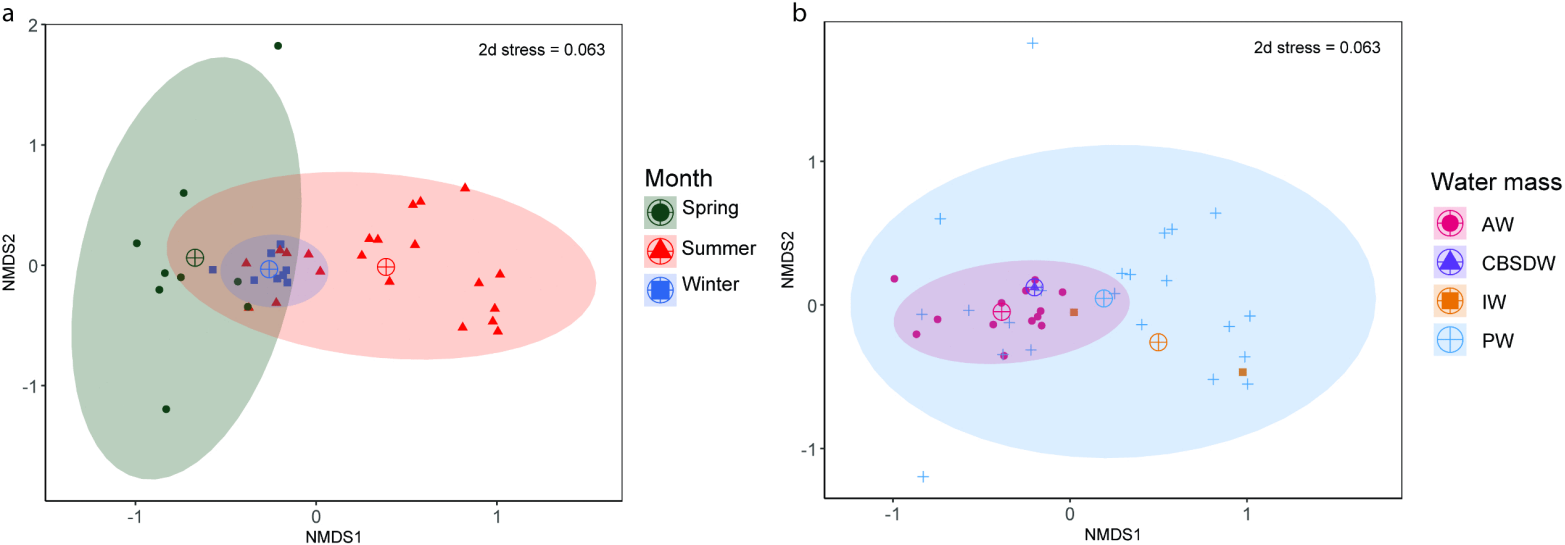
Macrozooplankton community comparison through non-metric multidimensional scaling (NMDS, k = 3 dimensions). a) Seasonal grouping of macrozooplankton communities. Stations sampled in spring are indicated in green, those sampled in winter are in blue, and those sampled in summer are in red. b) macrozooplankton communities grouped by water mass. Intermediate water in yellow, Atlantic water in red, Polar water in blue, Cold Barents Sea Dense water in purple. Group centroid is marked by a target symbol.

Macrozooplankton communities were not significantly different across water masses (ANOSIM: p = 0.34), but a pairwise comparison revealed that macrozooplankton communities in Atlantic waters were different to those in Polar waters (R = 0.14, p = 0.008). Macrozooplankton communities were significantly different across all seasons (ANOSIM: R = 0.32, p = 0.0003) (Fig 9a, 9b). Both community centroids as well as site dispersion distances were significantly different across water masses (PERMANOVA: F = 2.40, p = 0.03,PERMDISP: p = 0.0005) and season (PERMANOVA:F = 8.50 p = 0.01, PERMDISP: p = 0.002). As with fish community analyses, water mass classification was simplified to four groups (Polar waters, Atlantic waters, Intermediate water and Cold Barents Sea Dense water).

## Discussion

While we found significant differences in macrozooplankton community composition across seasons, no difference in pelagic fish community composition was measured across seasons or water masses. However, fish and macrozooplankton abundances estimated from acoustic transects were significantly correlated with distance from the Polar Front and ice edge as well as with temperature and salinity across all seasons. The considerable differences in the distribution of fish and macrozooplankton among the three seasons highlight the importance of multi-season studies, particularly for understanding the ecological impacts of static or semi-static oceanographic features such as the Polar Front.

### The Polar Front as a refuge for macrozooplankton in the spring

Pelagic fishes and macrozooplankton appeared to be most influenced by abiotic drivers, and while no statistical significance was measured in community composition across water masses, temperature was one of the most significant explanatory variables of fish and macrozooplankton distributions. Temperature has been shown to strongly influence fish and zooplankton biology as well as distribution in the Barents Sea [77–80]. This was most evident in this study during spring, when fishes, primarily occupied warmer Atlantic and modified Atlantic water and were nearly absent where Polar waters became dominant. This was observed in both geographic extent as well as vertically, with fish schools occupying deeper depths away from the cold surface Polar waters. The pelagic fish community during the spring survey was largely dominated by capelin and the observed distributional pattern is primarily attributed to that species.

Temperature preferences of capelin are highly dependent on body condition, age, food availability, among other parameters [81]. Adult capelin are able to withstand temperatures down to −1.8 °C, though the preferred range of temperature is between −1 °C and 3 °C, while juvenile capelin have a more limited range with a preference for warmer waters > 2 °C [14,31]. The vast majority of capelin caught during the spring survey were young and immature (< 13 cm standard length), and many of the adults caught were thin with empty stomachs [35], further explaining the presumed thermal constraints in the distribution of fish observed during the spring.

Capelin are known for following retreating sea ice to feed on the zooplankton that, in turn, follow phytoplankton blooms associated with the melting and retreating ice [14,17], but the species lack of antifreeze proteins limits their proximity to sea ice [82,83]. In spring, fish abundance was high at the commencement of the transect until about 75.6 ^o^N, where stratification of warm Polar and Polar water was present. In contrast, macrozooplankton abundance, biomass and diversity were low at the commencement of the transect and only began to increase at around 75.8 ^o^N and continued to increase as fish abundance decreased.

Macrozooplankton NASC was significantly negatively correlated with distance of the subsurface front and the daily ice edge, both found at the northern part of the transects. Based on Tucker trawl catches, macrozooplankton diversity increased with distance from the surface front and the one zooplankton group to increase in biomass with latitude was the mixed zooplankton group. This group was primarily composed of copepods of the genus *Calanus* that make up a large component of juvenile capelin diet [31,84]. These findings are consistent with the elevated secondary production and biomass of large copepods in cold waters north of the Polar Front [85], suggesting favorable feeding conditions for capelin were present at higher latitudes near the subsurface front. Based on findings from Basedow et al. in review, in spring mesozooplankton were most abundant in warm Polar water below 50 m, between 75.8 to 76.8 ^o^N. This finding coincides with the strong sound scattering layer at the 120 kHz frequency measured between 50–100 m in warm Polar water, where acoustic data suggests abundance of macrozooplankton without the presence of fishes. Similar cold water productive clusters have also been observed in the eastern portions of the Barents Sea Polar Front, where mesozoplankton and merozooplankton abundance and biomass were also linked to low temperatures and high latitudes [80,86].

Below the cold surface layer, acoustic signals from macrozooplankton did overlap with that of fish. Stomach content analysis of capelin during the spring survey showed overall low stomach fullness (0–10%) along the transect. Stomach fullness was higher just south of the Polar Front where fewer capelin were caught (40–60% fullness) [35]. Top-down control from capelin has been well documented in the region [24,87]. Most recently, Renaud et al. (2024) suggested top-down control as one possible contributor for the observed mismatch of primary and secondary production in the Barents Sea in two consecutive spring surveys, including during the spring 2022 survey analyzed in this study. The high abundance of macrozooplankton and mesozooplankton in cold waters during spring could thus be a result of the Polar waters, including the transformed warm Polar waters, providing a refuge from capelin grazing for those zooplankton species able to withstand cold waters.

In contrast to capelin, polar cod can resist colder temperatures, due to the presence of antifreeze protein and higher salt in serum [88], although the species is metabolically better suited for temperatures > 2 °C [89–91], and in the absence of predators or competition likely prefers waters above 5 °C [92]. During the spring survey, polar cod was more abundant in modified Atlantic waters with capelin and not in the warm Polar and Polar water where mesozooplankton and macrozooplankton were abundant. However, unlike capelin, polar cod were feeding more frequently and likely not as constrained by cold water. It has been suggested that the impact of major predators like capelin and polar cod is variable depending on water mass type in the Barents Sea [80], possibly explaining the cold Polar water zooplankton refugia observed in spring.

### Fish and macrozooplankton use of sea ice and the marginal ice zone

The distribution and abundance of pelagic species across the Polar Front was highly seasonal but stronger distributional patterns appeared during seasons with sea ice (spring and winter). The combination of strong seasonal changes in the Arctic and the lack of information about Arctic oceanic fronts across temporal scales results in our limited understanding of how the Polar Front and the ice edge can jointly influence pelagic ecology.

In winter, fish biomass was significantly higher under sea ice than in open Atlantic waters. The strong acoustic backscatter measured at the Polar Front and the ice edge was likely due to dense schools of polar cod. Dense under-ice schools of the species have been documented in the Canadian Arctic [93,94], and while no nets could be deployed to verify the acoustic signal, it is likely that polar cod was responsible for those dense aggregations due to the species life history, tolerance of freezing temperatures, and its presence in net catches further south. Overwintering strategies of polar cod are still not fully understood [30] but these observations support the current shift in our understanding of Arctic winter ecology as they provide insight into how active pelagic species, such as polar cod, distribute during the Polar Night. Recent studies have shown that pelagic activity during winter does not slow as previously believed and many pelagic species, including polar cod, feed, reproduce, and perform diel vertical migration during winter months [95–98]. Shallow water pelagic fish NASC was found to be the highest ranked variable in the random forest models used in this study for both macrozooplankton and deep water fish NASCs, suggesting that these under ice aggregations could be an ecologically important feature of the Barents Sea Polar Front in winter.

The distribution of macrozooplankton and fishes in spring and winter showed opposing patterns: in winter fish abundance increased with latitude and low temperatures while macrozooplankton abundance decreased. During spring, macrozooplankton abundance increased with latitude towards the subsurface Polar Front while fish abundance decreased. Macrozooplankton biomass distribution has been shown to be related to water masses in the Barents Sea [79,96]. In line with this, we observed higher densities of macrozooplankton in Polar and warm Polar waters in spring, but not in winter or summer. Predator-prey interactions are also likely to impact the distribution of macrozooplankton [24,87], with macrozooplankton abundance negatively correlated to pelagic fish abundance in spring.

The Barents Sea Polar Front surface conditions in the top 100 m are subject to more seasonal variation than at depth [3], so less variation in fish and macrozooplankton distribution across seasons would be expected at depth than at the surface. In summer, when seasonal ice was absent, fish abundance in the top 150 m were not correlated to any hydrographic features of the Polar Front. Only a few statistically significant, but weak, correlations were found among macrozooplankton NASC and environmental features related to the Polar Front, primarily surface and bottom temperatures. Conversely, in summer and spring, fish abundance below 150 m was significantly correlated to most hydrographic features of the Polar Front. The higher stability of the Polar Front at depth and the persistence of specific water masses and their associated could be what is creating a more pronounced correlation between fish abundance and the hydrographic features of the Polar Front.

Overall, summer was the only season when fish abundance below 150 m did not reflect patterns in fish abundance in the top 150 m. During summer, a warm surface layer and a strong thermal gradient forms due to atmospheric warming, stratifying the water column [99,100]. Strong vertical stratification has been shown to limit fish and zooplankton movement [61], and thus could be reducing connectivity between water masses during summer and would explain the differences in fish abundance patterns between shallow and deep waters. While surface water temperatures measured during summer were warm (5.1^o^ - 7.9 °C) and lacked the strong thermal gradient that was measured in spring and winter, a strong salinity gradient was still present. Kolodziejczyk et al. [100] recently suggested that in summer, the Barents Sea Polar Front is better defined by the sea surface salinity gradient as opposed to the thermal gradient that is often used [9,35,100]. The intensity of this summer time surface salinity gradient has been shown to be linked to freshwater input from the northern Barents Sea, and thus the strength of the Polar Front during summer is controlled by the extent of yearly ice cover [100,101]. It is predicted that with the loss of sea ice due to climate change this stratification will be reduced and Atlantic waters will become more prevalent, with vertical mixing extending throughout the entire water column even in summer [101]. The loss of water column stratification in the Barent’s Sea would likely mean the observed patterns in this study, and the ecological function of the Polar Front, may change dramatically as the climate keeps warming.

### Abundance of gelatinous zooplankton in summer

The dominance of gelatinous taxa in summer was remarkable but not unprecedented for the region [98,102]. Gelatinous zooplankton encompasses a diverse group of organisms, and despite their low energy content they can be a substantial food source for pelagic predators [103]. The distribution of gelatinous zooplankton is strongly influenced by water masses and bathymetry [104,105]. However, they are often excluded or under-reported in zooplankton surveys as their fragile bodies make them difficult to sample and identify [106]. Despite increased awareness of this sample bias, little is known about the distribution of gelatinous organisms in the Barents Sea in relation to the Polar Front. Recent studies from the Fram Strait [107,108] and Arctic Ocean [109] revealed a strong coupling between the presence of warmer Atlantic water masses and high abundance of certain gelatinous taxa. Accordingly, we only sampled abundant gelatinous zooplankton in those seasons with large presence of Atlantic waters, summer and winter. However, in the eastern Barents Sea large gelatinous zooplankton species were found to be associated with cold Arctic waters [80]. Gelatinous zooplankton association with specific water masses likely differs between life stage and further investigation of the ecology and distribution of gelatinous zooplankton in the Barents Sea is needed to understand their exact drivers.

## Conclusion

The influence of the Barents Sea Polar Front on pelagic communities appears to be important but highly variable across seasons. With the spring ice melt, pelagic fish appeared confined to warm water masses while macrozooplankton biomass and diversity increased in proximity of the subsurface Front. Contrastingly, in winter pelagic fish biomass increased considerably with proximity to both fronts (surface and subsurface) and the ice edge. In summer, fish and macrozooplankton biomass were the lowest although biodiversity was relatively high compared to spring. The Barents Sea Polar Front and its influence on pelagic communities is complex and difficult to generalize. Ecological studies in the Arctic commonly occur in summer months, as sea ice and winter weather can be prohibitive to fieldwork. This seasonal study has uncovered ecological patterns previously unknown, advancing our understanding of the Barents Sea Polar Front and emphasizing the need to consider seasonal variations in the ecology of pelagic organisms of the Barents Sea. The striking seasonal differences observed in pelagic fish and macrozooplankton communities in relation to the Polar Front highlight further the importance of multi-season work in understanding ecological patterns. Without seasonal information, management suffers from critical data gaps. We thus recommend increasing sampling from traditional and autonomous platforms during winter and spring in the Barents Sea to provide the best possible knowledge for sustainable management and resource use.

## Supporting information

S1 Table Net deployment details. (DOCX)

S2 Table Water stratification values. (DOCX)

S3 Table Fish diversity and biomass. (DOCX)

S4 Table Macrozooplankton diversity and biomass table. (DOCX)

S5 Table Random forest model parameters. (DOCX)

S1 Fig a-c Fig a. Log 38 kHz NASC above 150 m. b. Log 38 kHz NASC below 150 m. c. Log 120 kHz NASC. (TIF)

S2 Fig a-f Fig Temperature and salinity cross sections. (TIF)

S3 Fig 38 kHz NASC below 150 m map. (TIF)
