# Supplementary material for: Seasonal changes in the distributions of fish and zooplankton across the Barents Sea Polar Front
Source: PLoS One. 2026 May 11;21(5):e0348949. doi: 10.1371/journal.pone.0348949 (PMC13160360; doi:10.1371/journal.pone.0348949)
Supplement: S1 Table — (DOCX) [file pone.0348949.s001.docx]

**S1 Table. Net metadata**

| Site | Depth m | net depth m | Target depth | Speed knts | Net Type | ID | Date | Duration (hr) | Lat DD | Long DD | Vol. m^3 |
| --- | --- | --- | --- | --- | --- | --- | --- | --- | --- | --- | --- |
| 36 | 192 | 71 | 75 | 2.15 | Tucker | 36 | 06/01/24 | 0.17 | 77.36 | 30.21 | 679.1 |
| 74 | 345 | 249 | 250 | 2.96 | Pelagic | 74 | 07/01/24 | 0.33 | 75.52 | 29.52 | 162976.0 |
| 62 | 343 | 150 | 150 | 2.04 | Tucker | 62 | 07/01/24 | 0.17 | 75.50 | 29.52 | 586.5 |
| 63 | 345 | 251 | 250 | 2.17 | Tucker | 63 | 07/01/24 | 0.17 | 75.52 | 29.55 | 709.9 |
| 93 | 283 | 229 | 225 | 3.05 | Pelagic | 93 | 08/01/24 | 0.25 | 76.20 | 29.53 | 125936.0 |
| 84 | 279 | 122 | 120 | 2.03 | Tucker | 84 | 08/01/24 | 0.15 | 76.19 | 29.47 | 583.4 |
| 85 | 282 | 220 | 220 | 2.02 | Tucker | 85 | 08/01/24 | 0.17 | 76.21 | 29.53 | 617.3 |
| 117 | 257 | 128 | 130 | 2.75 | Pelagic | 117 | 09/01/24 | 0.29 | 76.76 | 29.58 | 116009.3 |
| 128 | 237 | 131 | 130 | 2.95 | Pelagic | 128 | 09/01/24 | 0.25 | 76.98 | 29.50 | 92600.0 |
| 108 | 253 | 112 | 100 | 1.98 | Tucker | 108 | 09/01/24 | 0.17 | 76.75 | 29.48 | 648.2 |
| 109 | 256 | 220 | 220 | 2.02 | Tucker | 109 | 09/01/24 | 0.15 | 76.76 | 29.55 | 527.8 |
| 164 | 243 | 153 | 140 | 2.04 | Tucker | 164 | 11/01/24 | 0.15 | 76.83 | 29.42 | 555.6 |
| 1098 | 368 | 233 | 300 | 3.11 | Pelagic | 1098 | 16/08/23 | 0.60 | 75.00 | 29.52 | 471148.8 |
| 1086 | 369 | 73 | 100 | 1.38 | Tucker | 1086 | 16/08/23 | 0.40 | 75.00 | 29.50 | 1037.1 |
| 1087 | 367 | 196 | 300 | 1.39 | Tucker | 1087 | 16/08/23 | 0.70 | 75.00 | 29.46 | 388.9 |
| 1088 | 366 | 133 | 180 | 1.38 | Tucker | 1088 | 16/08/23 | 0.50 | 75.00 | 29.40 | 1203.8 |
| 1128 | 332 | 183 | 180 | 3.09 | Pelagic | 1128 | 18/08/23 | 0.20 | 77.99 | 29.42 | 94822.4 |
| 1129 | 338 | 232 | 230 | 3.14 | Pelagic | 1129 | 18/08/23 | 0.30 | 78.03 | 29.50 | 128899.2 |
| 1112 | 329 | 94 | 40 | 1.61 | Tucker | 1112 | 18/08/23 | 0.30 | 78.01 | 29.48 | 722.3 |
| 1113 | 328 | 14 | 200 | 1.57 | Tucker | 1113 | 18/08/23 | 0.50 | 78.00 | 29.35 | 1389.0 |
| 1114 | 327 | 163 | 260 | 1.47 | Tucker | 1114 | 18/08/23 | 0.20 | 78.00 | 29.35 | 555.6 |
| 744 | 375 | 144 | 140 | 2.87 | Pelagic | 744 | 19/05/22 | 0.16 | 75.01 | 29.61 | 585.9 |
| 731 | 372 | 143 | 120 | 2.01 | Tucker | 731 | 19/05/22 | 0.20 | 75.01 | 29.59 | 693.6 |
| 732 | 372 | 250 | 250 | 1.92 | Tucker | 732 | 19/05/22 | 0.20 | 75.01 | 29.59 | 693.6 |
| 1149 | 229 | 102 | 140 | 3.00 | Pelagic | 1149 | 19/08/23 | 0.40 | 77.73 | 29.48 | 177792.0 |
| 1134 | 230 | 155 | 200 | 1.96 | Tucker | 1134 | 19/08/23 | 0.50 | 77.74 | 29.49 | 1018.6 |
| 1135 | 226 | 91 | 120 | 1.74 | Tucker | 1135 | 19/08/23 | 0.40 | 77.72 | 29.43 | 1926.1 |
| 1136 | 225 | 17 | 20 | 1.88 | Tucker | 1136 | 19/08/23 | 0.20 | 77.71 | 29.39 | 629.7 |
| 1169 | 189 | 28 | 40 | 2.71 | Pelagic | 1169 | 20/08/23 | 0.40 | 77.51 | 29.37 | 154086.4 |
| 1170 | 199 | 308 | 125 | 6.12 | Pelagic | 1170 | 20/08/23 | 0.40 | 77.54 | 29.42 | 177792.0 |
| 1154 | 189 | 132 | 150 | 1.80 | Tucker | 1154 | 20/08/23 | 0.50 | 77.50 | 29.43 | 1296.4 |
| 1155 | 186 | 481 | 25 | 4.46 | Tucker | 1155 | 20/08/23 | 0.60 | 77.52 | 29.36 | 1666.8 |
| 772 | 306 | 158 | 200 | 2.90 | Pelagic | 772 | 21/05/22 | 0.45 | 75.98 | 29.52 | 200016.0 |
| 767 | 291 | 116 | 115 | 1.74 | Tucker | 767 | 21/05/22 | 0.17 | 76.05 | 29.33 | 594.8 |
| 768 | 287 | 233 | 230 | 2.16 | Tucker | 768 | 21/05/22 | 0.18 | 76.05 | 29.34 | 636.3 |
| 1187 | 182 | 28 | 40 | 3.05 | Pelagic | 1187 | 21/08/23 | 0.50 | 77.38 | 29.48 | 274096.0 |
| 1188 | 190 | 98 | 120 | 3.36 | Pelagic | 1188 | 21/08/23 | 0.30 | 77.40 | 29.59 | 204460.8 |
| 1174 | 180 | 89 | 140 | 1.58 | Tucker | 1174 | 21/08/23 | 0.60 | 77.38 | 29.46 | 2222.4 |
| 1175 | 177 | 48 | 90 | 1.50 | Tucker | 1175 | 21/08/23 | 0.50 | 77.39 | 29.38 | 1203.8 |
| 1176 | 181 | 17 | 25 | 1.62 | Tucker | 1176 | 21/08/23 | 0.30 | 77.39 | 29.32 | 1111.2 |
| 781 | 281 | 76 | 100 | 3.05 | Pelagic | 781 | 22/05/22 | 0.38 | 75.49 | 29.56 | 147537.7 |
| 789 | 361 | 52 | 60 | 2.01 | Tucker | 789 | 22/05/22 | 0.19 | 75.46 | 29.77 | 674.9 |
| 790 | 361 | 21 | 120 | 2.27 | Tucker | 790 | 22/05/22 | 0.15 | 75.45 | 29.79 | 525.7 |
| 1218 | 192 | 91 | 100 | 3.22 | Pelagic | 1218 | 22/08/23 | 0.30 | 77.23 | 29.34 | 168902.4 |
| 1219 | 191 | 34 | 40 | 3.41 | Pelagic | 1219 | 22/08/23 | 0.30 | 77.25 | 29.47 | 177792.0 |
| 1200 | 191 | 32 | 40 | 1.82 | Tucker | 1200 | 22/08/23 | 0.40 | 77.25 | 29.48 | 814.9 |
| 1201 | 186 | 81 | 120 | 1.70 | Tucker | 1201 | 22/08/23 | 0.60 | 77.26 | 29.43 | 2222.4 |
| 805 | 369 | 108 | 140 | 2.92 | Pelagic | 805 | 23/05/22 | 0.47 | 74.98 | 29.01 | 155679.1 |
| 809 | 364 | 67 | 70 | 1.77 | Tucker | 809 | 23/05/22 | 0.28 | 74.99 | 29.06 | 1001.9 |
| 810 | 367 | 112 | 140 | 1.99 | Tucker | 810 | 23/05/22 | 0.30 | 74.98 | 29.08 | 1073.0 |
| 812 | 370 | 194 | 250 | 2.06 | Tucker | 812 | 23/05/22 | 0.39 | 74.95 | 29.12 | 1389.2 |
| 1241 | 226 | 63 | 100 | 3.21 | Pelagic | 1241 | 23/08/23 | 0.60 | 77.01 | 29.52 | 293356.8 |
| 1227 | 226 | 94 | 80 | 1.68 | Tucker | 1227 | 23/08/23 | 0.50 | 77.01 | 29.51 | 1296.4 |
| 1228 | 225 | 112 | 180 | 1.67 | Tucker | 1228 | 23/08/23 | 0.70 | 77.02 | 29.48 | 2074.2 |
| 848 | 207 | 27 | 25 | 2.08 | Tucker | 848 | 24/05/22 | 0.25 | 77.54 | 29.99 | 882.3 |
| 1260 | 254 | 71 | 90 | 3.30 | Pelagic | 1260 | 24/08/23 | 0.40 | 76.74 | 29.51 | 242982.4 |
| 1261 | 263 | 150 | 200 | 3.46 | Pelagic | 1261 | 24/08/23 | 0.40 | 76.78 | 29.51 | 260761.6 |
| 1250 | 253 | 115 | 200 | 1.65 | Tucker | 1250 | 24/08/23 | 0.80 | 76.75 | 29.52 | 2518.7 |
| 1251 | 253 | 65 | 100 | 1.64 | Tucker | 1251 | 24/08/23 | 0.50 | 76.73 | 29.53 | 1574.2 |
| 857 | 228 | 136 | 180 | 1.48 | Tucker | 857 | 25/05/22 | 0.83 | 77.04 | 29.54 | 2940.5 |
| 868 | 229 | 178 | 180 | 2.99 | Pelagic | 868 | 25/05/2024 | 0.52 | 76.97 | 29.80 | 237456.0 |
| 1283 | 277 | 169 | 220 | 3.40 | Pelagic | 1283 | 25/08/23 | 0.40 | 76.31 | 29.60 | 272614.4 |
| 1272 | 277 | 42 | 30 | 1.55 | Tucker | 1272 | 25/08/23 | 0.30 | 76.32 | 29.50 | 611.2 |
| 1273 | 276 | 125 | 220 | 1.84 | Tucker | 1273 | 25/08/23 | 0.20 | 76.31 | 29.48 | 814.9 |
