# Supplementary material for: Seasonal changes in the distributions of fish and zooplankton across the Barents Sea Polar Front
Source: PLoS One. 2026 May 11;21(5):e0348949. doi: 10.1371/journal.pone.0348949 (PMC13160360; doi:10.1371/journal.pone.0348949)
Supplement: S2 Table — (DOCX) [file pone.0348949.s002.docx]

**S2 Table. Water stratification values.**

| **N2 rad^2^/ s^2^** | **Stratification level** |
| --- | --- |
| <0 | Hydrostatically unstable |
| 0-.00002 | Non-stratified |
| 0.00002-.00005 | Weakly stratified |
| 0.00005 | Strongly stratified |
