# Supplementary material for: Seasonal changes in the distributions of fish and zooplankton across the Barents Sea Polar Front
Source: PLoS One. 2026 May 11;21(5):e0348949. doi: 10.1371/journal.pone.0348949 (PMC13160360; doi:10.1371/journal.pone.0348949)
Supplement: S3 Table — (DOCX) [file pone.0348949.s003.docx]

**S3 Table. Fish diversity and biomass.**

| Season | Site | Shannon Diversity Index | Simpsons Diversity Index | Density (fish/m3) | Number of spesies | Latitude (dd) | Longitude (dd) | Target Depth (m) | Water mass |
| --- | --- | --- | --- | --- | --- | --- | --- | --- | --- |
| August | 1098 | 1.21 | 0.65 | 0.01 | 5.00 | 75.00 | 29.52 | 300 | AW |
| August | 1283 | 0.66 | 0.31 | 0.00 | 5.00 | 76.31 | 29.60 | 220 | wPW |
| August | 1260 | 0.50 | 0.27 | 0.00 | 4.00 | 76.74 | 29.51 | 90 | wPW |
| August | 1261 | 0.68 | 0.33 | 0.00 | 5.00 | 76.78 | 29.51 | 200 | wPW |
| August | 1241 | 0.90 | 0.52 | 0.00 | 3.00 | 77.01 | 29.52 | 100 | wPW |
| August | 1242 | 0.00 | 0.00 | 0.02 | 1.00 | 77.03 | 29.65 | 180 | wPW |
| August | 1218 | 0.44 | 0.26 | 0.02 | 3.00 | 77.23 | 29.34 | 100 | PW |
| August | 1219 | 0.32 | 0.12 | 0.00 | 5.00 | 77.25 | 29.47 | 40 | wPW |
| August | 1187 | 0.06 | 0.02 | 0.01 | 3.00 | 77.38 | 29.48 | 40 | wPW |
| August | 1188 | 0.10 | 0.04 | 0.00 | 3.00 | 77.40 | 29.59 | 120 | PW |
| August | 1169 | 0.07 | 0.02 | 0.01 | 6.00 | 77.51 | 29.37 | 40 | wPW |
| August | 1170 | 0.02 | 0.01 | 0.00 | 5.00 | 77.54 | 29.42 | 125 | wPW |
| August | 1149 | 0.38 | 0.20 | 0.01 | 3.00 | 77.73 | 29.48 | 140 | wPW |
| August | 1128 | 0.52 | 0.32 | 0.21 | 6.00 | 77.99 | 29.42 | 180 | PW |
| August | 1129 | 0.79 | 0.52 | 0.02 | 4.00 | 78.03 | 29.50 | 230 | PW |
| January | 74 | 0.83 | 0.46 | 0.02 | 4.00 | 75.52 | 29.52 | 250 | AW |
| January | 93 | 0.95 | 0.51 | 0.06 | 6.00 | 76.20 | 29.53 | 225 | wPW |
| January | 117 | 0.02 | 0.00 | 0.84 | 7.00 | 76.76 | 29.58 | 130 | wPW |
| January | 128 | 0.61 | 0.30 | 0.03 | 6.00 | 76.98 | 29.50 | 130 | wPW |
| May | 805 | 0.00 | 0.00 | 0.40 | 1.00 | 74.98 | 29.01 | 105.00 | AW |
| May | 744 | 0.00 | 0.00 | 0.05 | 2.00 | 75.01 | 29.57 | 97.50 | AW |
| May | 781 | 0.07 | 0.03 | 0.63 | 2.00 | 75.49 | 29.56 | 80.00 | mAW |
| May | 772 | 0.32 | 0.15 | 0.07 | 3.00 | 75.98 | 29.52 | 159.00 | mAW |
| May | 868 | 0.18 | 0.08 | 0.05 | 3.00 | 76.97 | 29.80 | 180.00 | wPW |
