# Supplementary material for: Seasonal changes in the distributions of fish and zooplankton across the Barents Sea Polar Front
Source: PLoS One. 2026 May 11;21(5):e0348949. doi: 10.1371/journal.pone.0348949 (PMC13160360; doi:10.1371/journal.pone.0348949)
Supplement: S4 Table — (DOCX) [file pone.0348949.s004.docx]

**S4 Table. Macrozooplankton diversity and biomass table.**

| Month | Site | Shannon_DI | Simpsons_DI | Biomass(g) | chao1 | Start_Lat | Start_Long | Target_depth | Watermass |
| --- | --- | --- | --- | --- | --- | --- | --- | --- | --- |
| August | 1087 | 0.89 | 0.53 | 0.012688 | 6.00 | 75.00 | 29.46 | 300 | AW |
| August | 1088 | 0.74 | 0.40 | 0.002354 | 6.00 | 75.00 | 29.40 | 180 | AW |
| August | 1112 | 0.95 | 0.56 | 0.001393 | 3.00 | 78.01 | 29.48 | 40 | PW |
| August | 1113 | 1.49 | 0.70 | 0.001368 | 7.00 | 78.00 | 29.35 | 200 | wPW |
| August | 1114 | 1.54 | 0.76 | 0.001305 | 6.00 | 78.00 | 29.35 | 260 | PW |
| August | 1134 | 0.78 | 0.49 | 0.001511 | 6.00 | 77.74 | 29.49 | 200 | PW |
| August | 1135 | 0.98 | 0.56 | 0.000157 | 7.00 | 77.72 | 29.43 | 120 | wPW |
| August | 1136 | 1.03 | 0.54 | 0.001340 | 4.00 | 77.71 | 29.39 | 20 | wPW |
| August | 1154 | 1.10 | 0.55 | 0.001999 | 7.00 | 77.50 | 29.43 | 150 | wPW |
| August | 1155 | 1.20 | 0.63 | 0.000258 | 5.00 | 77.52 | 29.36 | 25 | wPW |
| August | 1174 | 1.27 | 0.61 | 0.001555 | 9.00 | 77.38 | 29.46 | 140 | wPW |
| August | 1175 | 1.68 | 0.79 | 0.001813 | 7.00 | 77.39 | 29.38 | 90 | wPW |
| August | 1176 | 1.13 | 0.64 | 0.000274 | 4.00 | 77.39 | 29.32 | 25 | wPW |
| August | 1200 | 0.53 | 0.25 | 0.000360 | 4.00 | 77.25 | 29.48 | 40 | wPW |
| August | 1201 | 1.41 | 0.69 | 0.001129 | 6.00 | 77.26 | 29.43 | 120 | wPW |
| August | 1227 | 1.72 | 0.79 | 0.000469 | 7.00 | 77.01 | 29.51 | 80 | wPW |
| August | 1228 | 1.00 | 0.56 | 0.001667 | 7.00 | 77.02 | 29.48 | 180 | wPW |
| August | 1250 | 0.61 | 0.33 | 0.002119 | 7.00 | 76.75 | 29.52 | 200 | wPW |
| August | 1251 | 0.99 | 0.51 | 0.000430 | 6.00 | 76.73 | 29.53 | 100 | wPW |
| August | 1272 | 0.44 | 0.22 | 0.000423 | 3.00 | 76.32 | 29.50 | 30 | wPW |
| August | 1273 | 1.06 | 0.59 | 0.007454 | 6.00 | 76.31 | 29.48 | 220 | wPW |
| January | 36 | 1.23 | 0.67 | 0.002784 | 7.00 | 77.36 | 30.21 | 75 | PW |
| January | 62 | 1.21 | 0.68 | 0.005297 | 6.00 | 75.50 | 29.52 | 150 | AW |
| January | 63 | 1.04 | 0.57 | 0.012357 | 7.00 | 75.52 | 29.55 | 250 | AW |
| January | 84 | 1.13 | 0.67 | 0.004424 | 5.00 | 76.19 | 29.47 | 120 | wPW |
| January | 85 | 1.19 | 0.67 | 0.011008 | 7.00 | 76.21 | 29.53 | 220 | wPW |
| January | 108 | 1.22 | 0.68 | 0.007156 | 6.00 | 76.75 | 29.48 | 100 | wPW |
| January | 109 | 1.10 | 0.65 | 0.005373 | 6.00 | 76.76 | 29.55 | 220 | wPW |
| January | 164 | 1.12 | 0.65 | 0.003698 | 5.00 | 76.83 | 29.42 | 140 | wPW |
| May | 731 | 0.00 | 0.00 | 0.000070 | 1.00 | 75.01 | 29.59 | 120 | AW |
| May | 732 | 0.68 | 0.49 | 0.002592 | 2.00 | 75.01 | 29.61 | 250 | AW |
| May | 767 | 0.99 | 0.59 | 0.002303 | 4.00 | 76.05 | 29.33 | 115 | wPW |
| May | 768 | 0.72 | 0.45 | 0.004274 | 5.00 | 76.05 | 29.34 | 230 | mAW |
| May | 789 | 0.00 | 0.00 | 0.000268 | 1.00 | 75.46 | 29.77 | 60 | wPW |
| May | 790 | 0.00 | 0.00 | 0.000151 | 1.00 | 75.45 | 29.79 | 120 | wPW |
| May | 812 | 0.32 | 0.14 | 0.004666 | 4.00 | 74.95 | 29.12 | 250 | AW |
| May | 848 | 1.24 | 0.63 | 0.001899 | 6.00 | 77.54 | 29.99 | 25 | PW |
| May | 857 | 1.08 | 0.57 | 0.001801 | 7.00 | 77.04 | 29.54 | 180 | wPW |
