# Supplementary material for: Seasonal changes in the distributions of fish and zooplankton across the Barents Sea Polar Front
Source: PLoS One. 2026 May 11;21(5):e0348949. doi: 10.1371/journal.pone.0348949 (PMC13160360; doi:10.1371/journal.pone.0348949)
Supplement: S5 Table — (DOCX) [file pone.0348949.s005.docx]

**S5 Table. Random forest final parameters**

| Modeled variable | **Model Characteristics** | |  | **Training Data: Regression Diagnostics** | |  | **Validation Data: Regression Diagnostics** | |
| --- | --- | --- | --- | --- | --- | --- | --- | --- |
| Shallow Fish NASC, spring |  |  |  |  |  |  |  |  |
|  | Number of Trees | 100 |  | R-Squared | 0.949 |  | R-Squared | 0.761 |
|  | Leaf Size | 5 |  | Mean Absolute Error (MAE) | 45.664 |  | Mean Absolute Error (MAE) | 96.785 |
|  | Tree Depth Range | 11-19 |  | Symmetric Mean Absolute Percentage Error (SMAPE) | 0.102 |  | Symmetric Mean Absolute Percentage Error (SMAPE) | 0.15 |
|  | Mean Tree Depth | 14 |  | Root Mean Square Error (RMSE) | 85.504 |  | Root Mean Square Error (RMSE) | 177.842 |
|  | % of Training Available per Tree | 90 |  | p-value | 0 |  | p-value | 0 |
|  | Number of Randomly Sampled Variables | 2 |  | Standard Error | 0.01 |  | Standard Error | 0.037 |
|  | % of Training Data Excluded for Validation | 30 |  |  |  |  |  |  |
| Deep Fish NASC, spring | Number of Trees | 100 |  | R-Squared | 0.984 |  | R-Squared | 0.93 |
|  | Leaf Size | 5 |  | Mean Absolute Error (MAE) | 3.951 |  | Mean Absolute Error (MAE) | 9.183 |
|  | Tree Depth Range | 12-20 |  | Mean Absolute Percentage Error (MAPE) | 0.168 |  | Mean Absolute Percentage Error (MAPE) | 0.489 |
|  | Mean Tree Depth | 14 |  | Root Mean Square Error (RMSE) | 7.271 |  | Root Mean Square Error (RMSE) | 15.439 |
|  | % of Training Available per Tree | 100 |  | p-value | 0 |  | p-value | 0 |
|  | Number of Randomly Sampled Variables | 3 |  | Standard Error | 0.006 |  | Standard Error | 0.018 |
|  | % of Training Data Excluded for Validation | 30 |  |  |  |  |  |  |
| Zoo NASC, spring | Number of Trees | 100 |  | R-Squared | 0.974 |  | R-Squared | 0.937 |
|  | Leaf Size | 5 |  | Mean Absolute Error (MAE) | 2.071 |  | Mean Absolute Error (MAE) | 4.148 |
|  | Tree Depth Range | 11-22 |  | Symmetric Mean Absolute Percentage Error (SMAPE) | 0.167 |  | Symmetric Mean Absolute Percentage Error (SMAPE) | 0.204 |
|  | Mean Tree Depth | 15 |  | Root Mean Square Error (RMSE) | 4.79 |  | Root Mean Square Error (RMSE) | 8.698 |
|  | % of Training Available per Tree | 100 |  | p-value | 0 |  | p-value | 0 |
|  | Number of Randomly Sampled Variables | 3 |  | Standard Error | 0.007 |  | Standard Error | 0.016 |
|  | % of Training Data Excluded for Validation | 30 |  |  |  |  |  |  |

| Modeled variable | **Model Characteristics** |  |  | **Training Data: Regression Diagnostics** | |  | **Validation Data: Regression Diagnostics** | |
| --- | --- | --- | --- | --- | --- | --- | --- | --- |
| Shallow Fish NASC, summer |  |  |  |  |  |  |  |  |
|  | Number of Trees | 100 |  | R-Squared | 0.838 |  | R-Squared | 0.586 |
|  | Leaf Size | 5 |  | Mean Absolute Error (MAE) | 7.937 |  | Mean Absolute Error (MAE) | 14.491 |
|  | Tree Depth Range | 12-20 |  | Mean Absolute Percentage Error (MAPE) | 0.774 |  | Mean Absolute Percentage Error (MAPE) | 1.043 |
|  | Mean Tree Depth | 16 |  | Root Mean Square Error (RMSE) | 27.075 |  | Root Mean Square Error (RMSE) | 38.948 |
|  | % of Training Available per Tree | 80 |  | p-value | 0 |  | p-value | 0 |
|  | Number of Randomly Sampled Variables | 3 |  | Standard Error | 0.014 |  | Standard Error | 0.041 |
|  | % of Training Data Excluded for Validation | 30 |  |  |  |  |  |  |
| Deep Fish NASC, summer | Number of Trees | 100 |  | R-Squared | 0.896 |  | R-Squared | 0.828 |
|  | Leaf Size | 5 |  | Mean Absolute Error (MAE) | 6.853 |  | Mean Absolute Error (MAE) | 9.18 |
|  | Tree Depth Range | 10-10 |  | Mean Absolute Percentage Error (MAPE) | 0.543 |  | Mean Absolute Percentage Error (MAPE) | 1.116 |
|  | Mean Tree Depth | 10 |  | Root Mean Square Error (RMSE) | 31.84 |  | Root Mean Square Error (RMSE) | 20.221 |
|  | % of Training Available per Tree | 100 |  | p-value | 0 |  | p-value | 0 |
|  | Number of Randomly Sampled Variables | 3 |  | Standard Error | 0.009 |  | Standard Error | 0.027 |
|  | % of Training Data Excluded for Validation | 30 |  |  |  |  |  |  |
| Zoo NASC, summer | Number of Trees | 100 |  | R-Squared | 0.705 |  | R-Squared | 0.829 |
|  | Tree Depth Range | 10-20 |  | Mean Absolute Error (MAE) | 0.182 |  | Mean Absolute Error (MAE) | 0.268 |
|  | Mean Tree Depth | 15 |  | Symmetric Mean Absolute Percentage Error (SMAPE) | 0.377 |  | Symmetric Mean Absolute Percentage Error (SMAPE) | 0.383 |
|  | % of Training Available per Tree | 60 |  | Root Mean Square Error (RMSE) | 0.8 |  | Root Mean Square Error (RMSE) | 1.091 |
|  | Number of Randomly Sampled Variables | 2 |  | p-value | 0 |  | p-value | 0 |
|  | % of Training Data Excluded for Validation | 30 |  | Standard Error | 0.017 |  | Standard Error | 0.017 |

| Modeled variable | **Model Characteristics** | |  | **Training Data: Regression Diagnostics** | |  | **Validation Data: Regression Diagnostics** | |
| --- | --- | --- | --- | --- | --- | --- | --- | --- |
| Shallow Fish NASC, spring |  |  |  |  |  |  |  |  |
|  | Number of Trees | 100 |  | R-Squared | 0.828 |  | R-Squared | 0.761 |
|  | Leaf Size | 5 |  | Mean Absolute Error (MAE) | 304.018 |  | Mean Absolute Error (MAE) | 96.785 |
|  | Tree Depth Range | 7-14 |  | Mean Absolute Percentage Error (MAPE) | 0.879 |  | Mean Absolute Percentage Error (MAPE) | 0.15 |
|  | Mean Tree Depth | 10 |  | Root Mean Square Error (RMSE) | 1364.354 |  | Root Mean Square Error (RMSE) | 177.842 |
|  | % of Training Available per Tree | 70 |  | p-value | 0 |  | p-value | 0 |
|  | Number of Randomly Sampled Variables | 1 |  | Standard Error | 0.018 |  | Standard Error | 0.037 |
|  | % of Training Data Excluded for Validation | 30 |  |  |  |  |  |  |
| Deep Fish NASC, spring | Number of Trees | 100 |  | R-Squared | 0.874 |  | R-Squared | 0.662 |
|  | Leaf Size | 5 |  | Mean Absolute Error (MAE) | 23.582 |  | Mean Absolute Error (MAE) | 22.869 |
|  | Tree Depth Range | 7-14 |  | Mean Absolute Percentage Error (MAPE) | 0.325 |  | Mean Absolute Percentage Error (MAPE) | 0.32 |
|  | Mean Tree Depth | 9 |  | Root Mean Square Error (RMSE) | 39.439 |  | Root Mean Square Error (RMSE) | 33.781 |
|  | % of Training Available per Tree | 70 |  | p-value | 0 |  | p-value | 0 |
|  | Number of Randomly Sampled Variables | 1 |  | Standard Error | 0.021 |  | Standard Error | 0.065 |
|  | % of Training Data Excluded for Validation | 30 |  |  |  |  |  |  |
| Zoo NASC, spring | Number of Trees | 100 |  | R-Squared | 0.709 |  | R-Squared | 0.289 |
|  | Leaf Size | 5 |  | Mean Absolute Error (MAE) | 0.076 |  | Mean Absolute Error (MAE) | 0.172 |
|  | Tree Depth Range | 8-10 |  | Mean Absolute Percentage Error (MAPE) | 3.778 |  | Mean Absolute Percentage Error (MAPE) | 4.854 |
|  | Mean Tree Depth | 9 |  | Root Mean Square Error (RMSE) | 0.167 |  | Root Mean Square Error (RMSE) | 0.571 |
|  | % of Training Available per Tree | 60 |  | p-value | 0 |  | p-value | 0 |
|  | Number of Randomly Sampled Variables | 4 |  | Standard Error | 0.026 |  | Standard Error | 0.026 |
|  | % of Training Data Excluded for Validation | 30 |  |  |  |  |  |  |
